# Being moved by modern abstract art

**DOI:** 10.3389/fpsyg.2026.1720357

**Published:** 2026-02-13

**Authors:** Xiaohan Zhou, Helmut Leder

**Affiliations:** Faculty of Psychology, University of Vienna, Vienna, Austria

**Keywords:** aesthetic emotion, art appreciation, being moved, museum study, visual art, predicting emotions

## Abstract

Although being emotionally moved has been studied as a key dimension of appreciation in visual art research, it remains unclear whether this response truly reflects the distinct emotion of being moved and whether such a complex emotion can be reliably elicited by abstract art in the absence of curatorial text, which provides minimal pre-existing narrative cues for meaning construction. This study explored whether and how such artworks can evoke the emotion of being moved. We conducted three exploratory studies triangulating across methods and contexts. Study 1 assessed how often being moved is related to visual art by collecting autobiographical moving memories using a modified *Geneva Affective Questionnaire*. Study 2 explored the characteristics of being moved by paintings. Conducted at the Heidi Horten Collection in Vienna, participants completed surveys before and after exhibition visits. They nominated up to five paintings that moved them and rated each selected work on its art features, emotional dimensions, bodily sensations, understanding, elicitors, and prosocial tendencies. Building on this, Study 3 aimed to explore whether being moved could emerge naturally without any priming or explicit guidance. Conducted in the same exhibition, this study used a broad, non-directive survey to measure whole-exhibition-visit emotion profile, including being moved without highlighting it. The results showed that strongly moving autobiographical memories were rarely related to visual art, whereas ratings of being moved in nominated paintings and whole-exhibition visits spanned the full intensity scale. Correlation analyses showed that understanding, elicitors, prosocial tendencies, as well as sadness and arousal, were significantly associated with being moved by paintings. In contrast, low-level visual features that were frequently nominated, such as color and composition, were not predictive, whereas content and personal relevance emerged as meaningful correlates. These exploratory findings suggest that even when viewing paintings without clear narratives and in the absence of curatorial text, viewers can experience genuine, though typically moderate, emotions of being moved. Such emotions appear to depend primarily on viewer-centered meaning-making and interpretive engagement. This not only highlights the cognitive demands involved in being moved by visual art but also underscores lay audiences’ capacity to construct meaning during visual art appreciation.

## 1 Introduction

There are moments in life that leave an indelible mark on the soul—when a long-lost family member steps through the doorway after years of absence, when a silent act of compassion pierces the darkness of war, when a loved one whispers a final farewell in the stillness of a hospital room, or when we turn away from someone dear, knowing fate may never reunite us. Such moments are often accompanied by warmth in the chest, an increased heart rate, a lump in the throat, and tears, and are described as instances of the emotion of being moved (Cova and Deonna, 2014). When these themes appear in art, they can likewise evoke powerful responses in viewers and have inspired some of history’s most celebrated masterpieces. Indeed, “being moved” has been regarded as part of the trinity of aesthetic emotions (Konečni, 2005) and as a dimension of aesthetic appreciation (Vessel et al., 2012).

Despite increasing scholarly interest, the construct of being moved remains theoretically and empirically challenging. The difficulties arise from vague terminology and definitions (Seibt et al., 2017) as well as from its highly personal nature (Tokaji, 2003). A widely studied variant—here referred to as the emotion of being moved—is typically characterized as bittersweet: a dynamic admixture of sadness and joy whose intensity and outward expression vary across individuals depending on personal experiences, empathic capacity, and socially oriented dispositions such as attachment, communal sharing, or moral sensitivity (Kuehnast et al., 2014; Zickfeld et al., 2019a; Cova et al., 2017). Empirical findings suggest that the emotion of being moved recruits higher-level processes that integrate affective, cognitive, and social mechanisms in ways not yet fully specified (Menninghaus et al., 2015). Put succinctly, eliciting^1^ this emotion is complex: it depends on cognitive factors such as narrative richness and meaningfulness (Landmann et al., 2019), as well as individual predispositions including trait empathy (Eerola et al., 2016) and cultural background (Danning, 2023). Proposed elicitors include perceptions of moral goodness or virtue (Cova and Deonna, 2014), sudden intensifications of communal sharing (Fiske et al., 2017), cues of attachment marked by mixed positive–negative valence (Kuehnast et al., 2014), and recognition that something in the stimulus is exceptionally important or irreplaceable to the self (Cullhed, 2020). These accounts converge on the idea that the emotion of being moved often requires semantic elaboration or meaning construction to activate higher-order social-relational appraisals.

Visual art—especially paintings that provide minimal narrative cues for meaning construction—presents a distinct empirical challenge within this framework. Paintings are typically unimodal, relying primarily on the visual channel. In “low-narrative” contexts, which in this paper refers to abstract or conceptual works with little inherent narrative that are presented without curatorial text (e.g., creation stories, artist background, or explanations of meaning or value), viewers receive little explicit narrative information, particularly if they are non-experts. As a result, stand-alone low-narrative paintings may offer fewer cues for constructing social or moral meaning. Moreover, most museum visitors are not especially “diligent”: average viewing times are around 20–30 s per painting (Smith et al., 2017; Carbon, 2017) and about 20 min for an entire exhibition regardless of its size (Serrell, 1997). This contrast motivates the central research question of the present paper: can purely visual, low-narrative artworks presented without contextual or textual framing reliably elicit the complex emotion of being moved? If so, what are the characteristic emotional, bodily, and cognitive features of such responses to visual art, and how do they compare to being moved by other elicitors such as multimodal artforms (film, music) or autobiographical life experiences? More specifically, can being moved by visual art be regarded as a genuine emotion—comparable to the emotion identified in affective research—or merely as a general state of emotional intensity and aesthetic engagement?

To address these questions, we adopt a triangulation strategy combining autobiographical memory recall with naturalistic museum observation. First, we test whether visual features alone—in a low-narrative gallery setting—can evoke the multifaceted experience typically associated with the emotion of being moved. Second, we aim to characterize the phenomenology and candidate elicitors of visual-art-elicited being moved, and to compare these with being moved elicited by multimodal artworks and autobiographical events. In doing so, we seek to clarify the nature of being moved by visual art and to shed light on its role in aesthetic appreciation.

### 1.1 The emotion of being moved

Research in affective science has established being moved by certain prototypical factors—such as moral goodness and love (Hänninen and Koski-Jännes, 2025)—as a distinct emotional state (Cova and Deonna, 2014; Zickfeld et al., 2019a). Many studies have induced this state using thematically rich, multimodal stimuli such as film clips (Hanich et al., 2014), poetry (Wassiliwizky et al., 2017a), and music (Vuoskoski and Eerola, 2017) that depict core human experiences such as love, loss, reunion, or self-sacrifice. These materials provide narrative, social, and situational cues that readily support the appraisals and meaning constructions associated with the emotion being moved.

Physiological and behavioral markers commonly observed include chills, goosebumps, and pleasurable tears, while subjective reports frequently mention a warm sensation in the chest and an ambivalent blend of sadness and joy (Kuehnast et al., 2014; Wassiliwizky et al., 2017b; Zickfeld et al., 2019b). Although individual episodes can involve strong affective engagement, aggregate psychophysiological data often place overall arousal in the mid-to-low range. This supports the characterization of the emotion of being moved as a predominantly positive, mixed-emotion state rather than a purely high-arousal outburst (Kuehnast et al., 2014; Menninghaus et al., 2015). Autobiographical recall studies corroborate these findings: real-life moving events such as reunions, partings, births, and deaths typically show moderate arousal, warm bodily sensations, moist eyes, and sustained intensity (Bartsch et al., 2014; Zickfeld et al., 2019b). Importantly, the emotion of being moved shows consistent links to prosocial tendencies and social cognition—it correlates with empathy, compassion, and the inclination to help or connect with others. Some researchers therefore conceptualize it as a social–moral emotion that signals communal value or attachment (Fiske et al., 2019; Menninghaus et al., 2015).

In aesthetic contexts, the emotion of being moved also predicts evaluative outcomes: it is associated with greater liking, deeper appreciation, and increased enjoyment of otherwise sad or challenging content (Hanich et al., 2014; Wassiliwizky et al., 2015; Vuoskoski and Eerola, 2017). When audiences adopt an art schema—that is, when they frame stimuli as “art”—sadness can positively contribute to enjoyment, with the emotion of being moved often mediating this relationship (Menninghaus et al., 2019).

### 1.2 Being moved as an aesthetic-appreciation dimension

In empirical aesthetics, being moved is sometimes framed less as a distinct emotion than as an aesthetic-appreciation dimension: an intensification of the aesthetic episode characterized by heightened appreciation, sudden insight, or amplified affective intensity in response to the artwork’s perceptual properties (Vessel et al., 2013; Cabbai et al., 2024). Low-narrative paintings communicate mainly through visual form and often lack explicit narrative content. Viewers—especially lay audiences—may therefore need to invest more interpretive effort or possess background knowledge to extract social or moral meaning (Waligórska, 2006). When paintings are presented without explanatory labels, meaning construction becomes particularly central to deep emotional engagement.

Nevertheless, recent studies indicate that even brief encounters with paintings can produce strong reports of being moved, with perceptual durations as short as 6–8 s (Vessel et al., 2012, 2019; Cabbai et al., 2024). As predominantly unimodal stimuli, paintings provide a useful testbed: does being moved in visual-art studies correspond to the literal emotion of being moved? According to Vessel et al. (2012, 2013, 2019), ratings of being moved often reflect heightened visual appeal and intensification of overall emotional experience rather than the emotion itself^2^. Similarly, Gerger et al. (2018) and Cabbai et al. (2024) reported positive correlations between subjective arousal and self-reported movingness in painting encounters, a pattern contrasting with the mid-to-low arousal typical of being emotionally moved. These findings suggest that what is often assessed in visual-art contexts may not be the emotion of being moved but rather a more general “thrilled state” (Konečni, 2005) characterized by heightened overall emotional intensity.

### 1.3 Current research: overview and questions

Thus, being emotionally moved may encompass two related but distinct aspects: (1) the specific emotion of being moved, as described in affective research, and (2) the general intensification of emotional experience often reported in visual art studies, which does not necessarily constitute a unique emotion. Motivated by this putative distinction between the emotion of being moved and being emotionally moved as an aesthetic-judgment dimension, the present research investigates whether, and how, low-narrative paintings presented without explanatory text can elicit the complex and unique emotion of being moved. Low-narrative exhibitions provide a conservative and ecologically valid test: by minimizing external narrative scaffolding, they challenge the assumption that bittersweet emotional states necessarily require explicit narrative or multimodal cues. Studying lay viewers in such settings therefore allows us to explore whether the visual modality and interpretive engagement alone can generate an emotional profile consistent with the emotion of being moved as characterized in affective science.

We address these questions across three exploratory studies triangulating different methods and contexts. Studies 1 and 2 used autobiographical recall to capture prototypical moving experiences and to study the types of events participants nominated as their most moving, with particular attention to whether visual-art memories were included. Studies 2 and 3 were field studies conducted at the *KLIMT ⇄ WARHOL* exhibition in the Heidi Horten Collection, Vienna, where participants freely explored a low-narrative painting exhibition and provided ratings of both individual paintings and the whole exhibition. This mixed-method approach allows comparison of memory-based, multimodal, socially embedded moving experiences with in-situ, unimodal, visual-art-elicited experiences under naturalistic conditions.

Specifically, we ask three interrelated questions. First (RQ1), within a low-narrative exhibition context (at both the individual-painting and whole-visit levels), how likely are people to report being moved while viewing visual art? Second (RQ2), what are the characteristic features of being moved by paintings—including artistic, emotional, bodily, cognitive, elicitor-related and prosocial aspects—and which of these dimensions emerge as correlates? Third (RQ3), in what ways does being moved by paintings resemble or diverge from being moved in multimodal or autobiographical contexts across these dimensions?

Across these exploratory studies, we aim to clarify how the emotion of being moved manifests in encounters with visual art, particularly low-narrative paintings. If visual features and interpretive engagement suffice to elicit classic social-affective markers (e.g., warmth, moist eyes, prosocial impulses), this would suggest considerable overlap between autobiographical and art-elicited moving experiences. Conversely, if art-elicited movingness primarily reflects intensified affective intensity and aesthetic appreciation without robust prosocial markers, this would point to partially distinct pathways to being moved. Clarifying these possibilities will advance a more nuanced conceptualization of being moved and inform broader discussions of how artform, context, cognition, and personal relevance interact to construct this complex emotion in art perception.

## 2 Study 1

Study 1 served as an initial step to explore the prototypical structure of being moved experiences through autobiographical recall. Before turning to museum-based investigations, it was important to establish a baseline description of how people typically understand, label, and evaluate their own moving memories. This study therefore explored two core issues: first, whether different German terms for being moved, being moved (German: *bewegt*) and being stirred (German: gerührt) capture distinct nuances of the experience (Kuehnast et al., 2014), where the former refers to broader range of emotional states, including complex, aroused emotions such as sadness or awe, whereas the latter focuses more on warm, tender, or softly touched feeling; and second, whether social context (group vs. alone) influences the intensity and profile of recalled moving events. By analyzing the emotional components, elicitors, and event types reported, Study 1 provides a benchmark against which later art-related findings can be compared.

### 2.1 Methods

#### 2.1.1 Ethics

All procedures were carried out in accordance with the World Medical Association’s *Declaration of Helsinki* and with applicable professional ethical guidelines for psychological research. Study 1 was implemented as an addendum to an emotional-image experiment (facial electromyography and skin conductance; total session duration approximately 1 h) and received formal ethics approval from the Ethics Committee of the University of Vienna (Reference No. 01253). The approved protocol covered both the physiological experiment and the additional questionnaire reported here. Participants in Study 1 provided written informed consent prior to participation; this consent covered both the physiological measures, and the questionnaire measures described below.

#### 2.1.2 Participants

Twenty-two participants (median age = 26; age range 19–67; 18 female, 1 preferred not to disclose) were recruited via the Laboratory Administration for Behavioral Sciences (LABS). All participants reported normal or corrected-to-normal vision and intact color perception. Participants provided written informed consent and received €20 as compensation (paid together with participation in the accompanying emotional-image experiment). Experimental tasks were performed with each participant’s dominant hand; for the questionnaire portion the skin-conductance electrode on the non-dominant hand was removed so that participants could complete the pencil-and-paper questionnaire comfortably.

#### 2.1.3 Questionnaire and procedure

Following the emotional-image experiment, participants firstly completed a set of questionnaires to assess individual differences and baseline affect. The battery comprised: art interest measured by the VAIAK–Interest scale (Vienna Art Interest and Art Knowledge Questionnaire; Specker et al., 2020), self-reported frequency of art engagement, baseline affect using the PANAS (Positive and Negative Affect Schedule; Crawford and Henry, 2004), and empathy via the Empathic Concern subscale of the QCAE (Questionnaire of Cognitive and Affective Empathy; Reniers et al., 2011).

Subsequently, participants completed a questionnaire based on the German version of the *Geneva Appraisal Questionnaire* (GAQ; https://www.unige.ch/cisa/research/materials-and-online-research/research-material/) and was modified to capture features of being moved (see Supplementary material). Participants were asked to recall autobiographical memories of emotionally moving experiences and to report these memories in two contexts: when other people were present (group context) and when they were alone (alone context). This manipulation was included to explore how social interaction influences the moving experience.

For each recalled episode participants provided a brief written description and then completed closed-format ratings. Core emotional dimensions included ratings of being moved (German: *bewegt*) and being stirred/touched (German: *gerührt*), together with standard affect items (happiness, sadness), overall valence, arousal, and avoidance — all rated on 7-point Likert scales. Participants also indicated the main factor that made the event moving and rated a set of hypothesized elicitor items (for example: dearness, irreplaceability, perceived unacceptable loss, relationship closeness, and attachment). The discrete-emotion checklist was adapted from prior being-moved research: items judged irrelevant to being moved (e.g., contempt, despair) were removed and items considered relevant to moving episodes (helplessness, relief, embarrassment, fascination) were added. Participants could select multiple discrete emotions for each recalled episode.

The questionnaire was administered as the final part of an emotional-image experiment, where participants viewed paired images (happy and sad) and rated how moving each pair felt. There was no time limit for completing the memory questionnaire, and participants were free to ask the experimenter for clarification.

### 2.2 Results and discussion

#### 2.2.1 Emotional intensity

**Figure 1:**
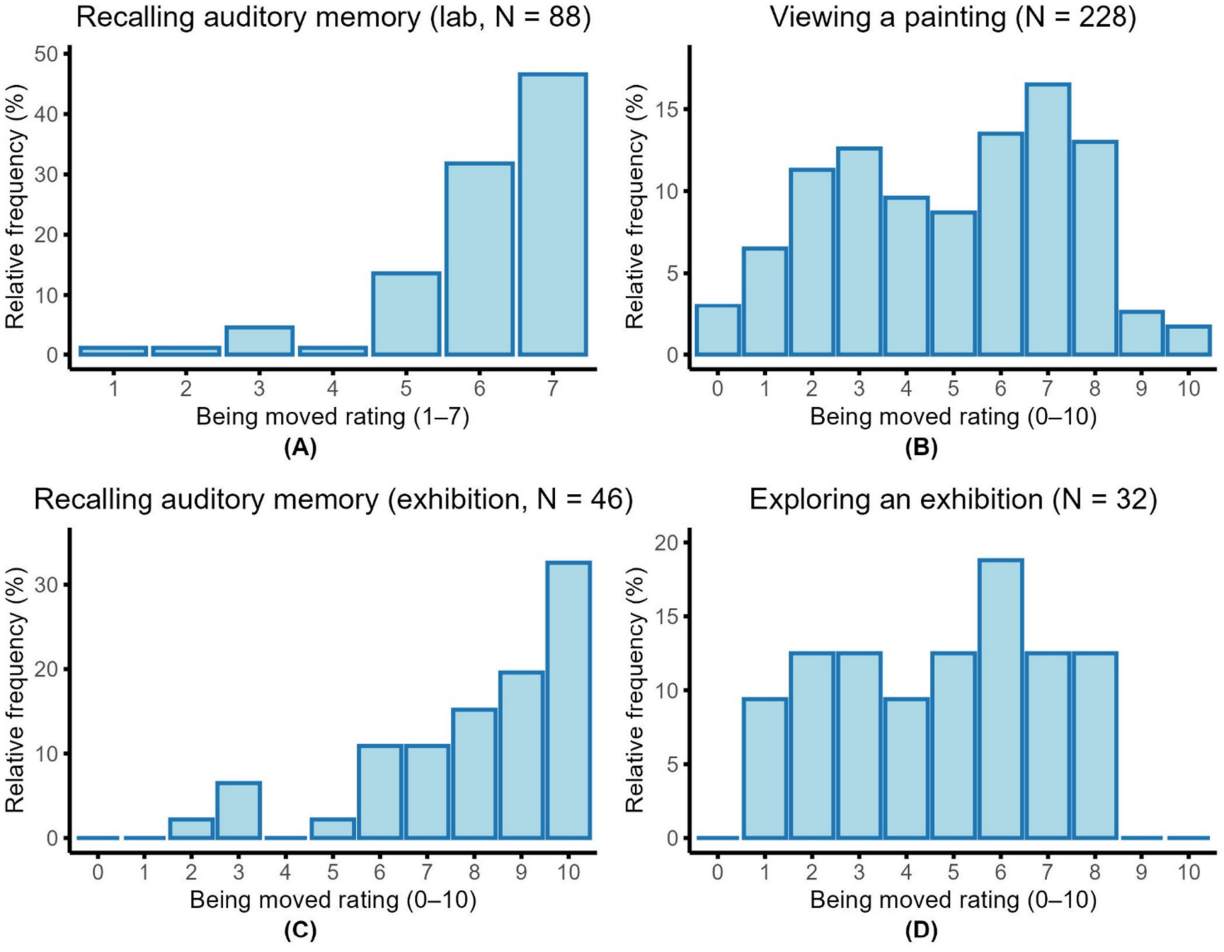
Distributions of self-reported being-moved ratings across tasks and studies. **(A)** Recalling an autobiographical memory in the lab (Study 1). **(B)** Viewing a painting during the museum visit (Study 2). **(C)** Recalling an autobiographical moving memory in the exhibition context (Study 2). **(D)** Exploring the exhibition as a whole (Study 3). Relative frequencies (%) are shown on the *y*-axis. Ratings were given on 7-point **(A)** or 10-point **(B–D)** scales.

Intensity ratings were consistently high (see Supplementary Table 1; Figure 1A). In the group context, both terms had a median of 7 on the 1–7 scale (*bewegt* IQR = 1; *gerührt* IQR = 1.75). In the alone context, both medians were 6 (*bewegt* IQR = 1; *gerührt* IQR = 1.75). In the group condition, 54.5% of responses reached the maximum rating for both terms. In the alone condition, this decreased to 40.9% (*bewegt*) and 36.4% (*gerührt*), though overall intensity remained high.

To explore potential effects of context and terminology, we conducted a 2 (Context: group vs. alone) × 2 (Terminology: *bewegt* vs. *gerührt*) nonparametric repeated-measures ANOVA using the aligned rank transform, which does not assume normality and is more appropriate for our small sample size. No significant main effects emerged for context, *F* (1, 63) = 0.86, *p* = 0.36, partial η^2^ = 0.04, or terminology, *F* (1, 63) = 0.51, *p* = 0.48, partial η^2^ = 0.02, and no significant interaction was found, *F* (1, 63) = 0.19, *p* = 0.66, partial η^2^ = 0.01. To further assess convergence between the two terms, we used Spearman correlations for the same reason (non-normality). Strong associations were observed between *bewegt* and *gerührt* in both contexts (group: *ρ* = 0.669; alone: ρ = 0.696; both ps < 0.001). Overall rating across two contexts are shown in Supplementary Table 2.

These results suggest that autobiographical recall robustly elicits intense moving experiences. Emotional intensity did not significantly differ across social contexts or terminology, contrasting with the proposal that being moved is primarily a social-relational emotion triggered by communal sharing (Fiske et al., 2019). Our findings instead indicate that solitary experiences can be equally intense. The strong correlations between *bewegt* and *gerührt* support their conceptual overlap, consistent with Kuehnast et al. (2014) and Menninghaus et al. (2015), who treat both as expressions of the same overarching “being moved” dimension, translated in English as being moved or being stirred, but differentiated in German nuances such as a broader, more aroused emotional state (*bewegt*) versus a warmer, softer, tender tone (*gerührt*). Although distributions suggest that group contexts may slightly amplify intensity, social interaction is not a necessary condition.

#### 2.2.2 Eliciting events

To categorize the types of recalled events, two independent raters classified each memory using an adapted framework from Menninghaus et al. (2015). After reconciliation, four categories were identified (see Table 1): relationship events (50.0%), critical life experiences (29.5%), art-related events (15.9%), and miscellaneous experiences (4.5%).

**Table 1:** Types and subtypes of moving events recalled in Study 1 and Study 2.

Event type	Event subtype	Study 1		Study 2	
		*N*	%	*N*	%
Relationship	family bonding	6	27.3	3	16.7
	Friendship	4	18.2	6	33.3
	helping others	3	13.6	1	5.6
	Reunion	4	18.2	0	0.0
	romantic love	4	18.2	1	5.6
	Separation	1	4.5	5	27.8
	mics.	0	0.0	2	11.1
	Overall	**22**	**50.0**	**18**	**39.1**
Critical life	birth & pregnancy	0	0.0	2	22.2
	death & funeral	2	15.4	3	33.3
	Illness	4	30.8	0	0.0
	psychological difficulty	1	7.7	2	22.2
	self-care	1	7.7	0	0.0
	self-reflection	1	7.7	0	0.0
	Success	2	15.4	2	22.2
	mics.	2	15.4	0	0.0
	Overall	**13**	**29.5**	**9**	**19.6**
Art-related	Episode	1	14.3	0	0.0
	Movie	3	42.9	3	27.3
	museum & collection	0	0.0	2	18.2
	Painting	0	0.0	1	9.1
	performance & theater & opera	0	0.0	3	27.3
	Photo	0	0.0	1	9.1
	song & chorus	3	42.9	1	9.1
	Overall	**7**	**15.9**	**11**	**23.9**
Mics.	experience & beauty of nature	0	0.0	7	87.5
	mics.	2	100.0	1	12.5
	Overall	**2**	**4.5**	**8**	**17.4**
Overall		**44**	**100.0**	**46**	**100.0**

Bold values within each event type indicate the total number and the percentage of the entire dataset, including all event types in each study.

Relationship events included family bonding (27.3%), friendship (18.2%), reunions (18.2%), and romantic love (18.2%). Critical life experiences encompassed illness, loss, and major personal challenges. Only 7 out of 44 events (15.9%) were art-related, with most involving film (42.9%) or music/choir performances (42.9%). No participants identified purely visual art (e.g., painting, photography, visual art exhibition visits) as their most moving life event.

These findings replicate Menninghaus et al. (2015), who also found that only a small minority of moving events were art-related and none were purely visual artworks. While art can certainly evoke strong responses in experimental or curated settings, such experiences appear to be rarely recalled as the most emotionally impactful life events. This suggests that although art-related activities can evoke moving experiences, deeply moving autobiographical memories are more often rooted in social or personally significant contexts.

#### 2.2.3 Emotional profiles and elicitors

Ratings of valence, arousal, emotional components, and elicitors (see Supplementary Table 2) showed consistently high scores for *bewegt*, *gerührt*, and arousal (medians = 5–6; IQRs = 1–3), whereas sadness had a lower median rating (2.5) and a wider distribution (IQR = 4.25). The most frequent emotional components were joy (65.9%), relief (52.3%), and sadness (47.7%), followed by pleasure (27.3%) and helplessness (27.3%). Less common were pride (22.7%), fascination (18.2%), and guilt (11.4%). Fear, disgust, and anger were rare (<10%). Only one participant reported no emotional component.

As shown in Supplementary Tables 2, 3, Spearman analyses revealed a significant correlation between *bewegt* and arousal in the alone context [*ρ* = 0.602, *p* = 0.055, false discovery rate (FDR) corrected] and overall (ρ = 0.531, *p* < 0.001). Elicitors such as dearness, irreplaceability, relationship closeness, and attachment were rated relatively high (medians = 5–6), while sadness/unacceptability received slightly lower ratings (median = 5).

As shown in Supplementary Tables 2, 4. Comparisons across event types showed distinct profiles. Art-related memories were associated most with sadness (71.4%), joy (42.9%), and relief (42.9%). Relationship memories were primarily linked to joy (77.3%) and relief (50.0%). Critical life events showed higher frequencies of anxiety (46.2%), helplessness (30.8%), fear (23.1%), and guilt (15.4%). Relationship and life-event memories received especially high elicitor ratings (medians = 5–7). Art-related memories, by contrast, showed lower ratings for relationship closeness (median = 4.5) and attachment (median = 5), though they were still rated relatively high in dearness and irreplaceability. No strong correlations were found between *bewegt* or *gerührt* and other emotion dimensions or elicitors.

Taken together, these results highlight the complex, ambivalent structure of moving autobiographical memories. Joy and sadness frequently co-occurred, consistent with prior findings (Zickfeld et al., 2019b; Schindler et al., 2022). Relief also played a significant role, aligning with Tokaji’s (2003) model that emphasized relief and tension as central mixed emotions in being moved—though tension was not measured here. While art-related memories were less socially grounded, they still showed high ratings for dearness, suggesting that strong moving experiences do not necessarily require interpersonal cues. Differences between *bewegt* and *gerührt* correlations indicate that *bewegt* may capture a more internally activating emotional state, whereas *gerührt* may be more tied to interpersonal meaning.

### 2.3 Summary

Study 1 explored the emotional profile and perceived elicitors of being moved using an autobiographical recall paradigm that explicitly compared two German terms (*bewegt* and *gerührt*) and two social contexts (group vs. alone). Standardized to a 0–10 scale, participants showed very high art-activity frequency (median 10.0, range 0.0–10.0), moderate Empathic Concern (median 6.7, range 2.5–9.2), moderate art interest (median 5.2, range 0.2–9.4), and comparatively low affect on PANAS: Positive Affect median 3.1 (range 0.5–7.5) and Negative Affect median 2.3 (range 0.0–4.3). More details—including original (unstandardized) scale scores and correlation analyses between being moved and individual-difference measures—are provided in the Supplementary Table 11. Participants consistently reported intense moving experiences across both contexts and terms. Intensity did not significantly differ by condition, although group contexts showed somewhat higher peak responses.

Recalled events were dominated by relationship and critical life experiences, with only a minority classified as art-related and none involving purely visual artworks. This suggests that deeply moving autobiographical memories are unlikely to stem from visual art alone.

Emotional responses were complex and ambivalent: joy, relief, and sadness co-occurred frequently. Elicitor ratings emphasized dearness, irreplaceability, and attachment as central to being moved, supporting the emotion’s characterization in prior studies (e.g., Cullhed, 2020).

Overall, although the small sample size limits the generalizability, this study demonstrates that people asked to recall autobiographical moving memories consistently report experiencing the emotion of being moved, providing a descriptive baseline for later studies. The findings suggest that while art may evoke meaningful experiences, the strongest and most prototypical moving memories are rooted in socially and personally significant life events.

## 3 Study 2

### 3.1 Methods

Study 1 provided evidence that autobiographical recall reliably elicits strong moving experiences, typically rooted in social or personally significant life events, with only a small fraction of art-related memories and virtually none from purely visual artworks. Building on this baseline, Study 2 shifts focus to real-time encounters with paintings in a museum setting. The aim was to explore whether visual art—particularly low-narrative paintings presented without interpretive text—can elicit the emotion of being moved in everyday viewing conditions. Unlike Study 1, which relied on memory recall, Study 2 used in-situ field data collected during unguided exhibition visits, allowing us to capture immediate phenomenological, bodily, and cognitive features of being moved by visual art.

#### 3.1.1 Ethics

Studies 2 and 3 were conducted as field-study components of a Master’s-level psychology course at the Department of Psychology, University of Vienna. Both took place at the permanent exhibition of the Heidi Horten Collection, where all displayed artworks were selected by public vote and did not include violent, graphic, or other distressing content. Both studies made exclusive use of completely anonymous questionnaires and involved only minimal-risk behavioral measures (unguided exploration of the exhibition and subsequent online ratings). Participants were informed about the tasks they were asked to perform, the anonymity of the data, the voluntary nature of participation, and their right to withdraw at any time. Participation was indicated via an explicit agreement item at the start of the online questionnaire; in keeping with provisions permitting dispensation of documented written consent for anonymous, minimal-risk questionnaire studies, no separate written consent forms were recorded for Studies 2 and 3. No identifying information was collected, and all participants included in the analyses were over 18 years of age.

#### 3.1.2 Participants

Forty-six participants (median age = 24; age range 21–61; 31 female) took part in Study 2. The sample consisted primarily of Master’s psychology students and their friends or family who participated as part of a course field activity. Participants received free admission to the Heidi Horten Collection for their participation.

#### 3.1.3 Exhibition setting

**Figure 2:**
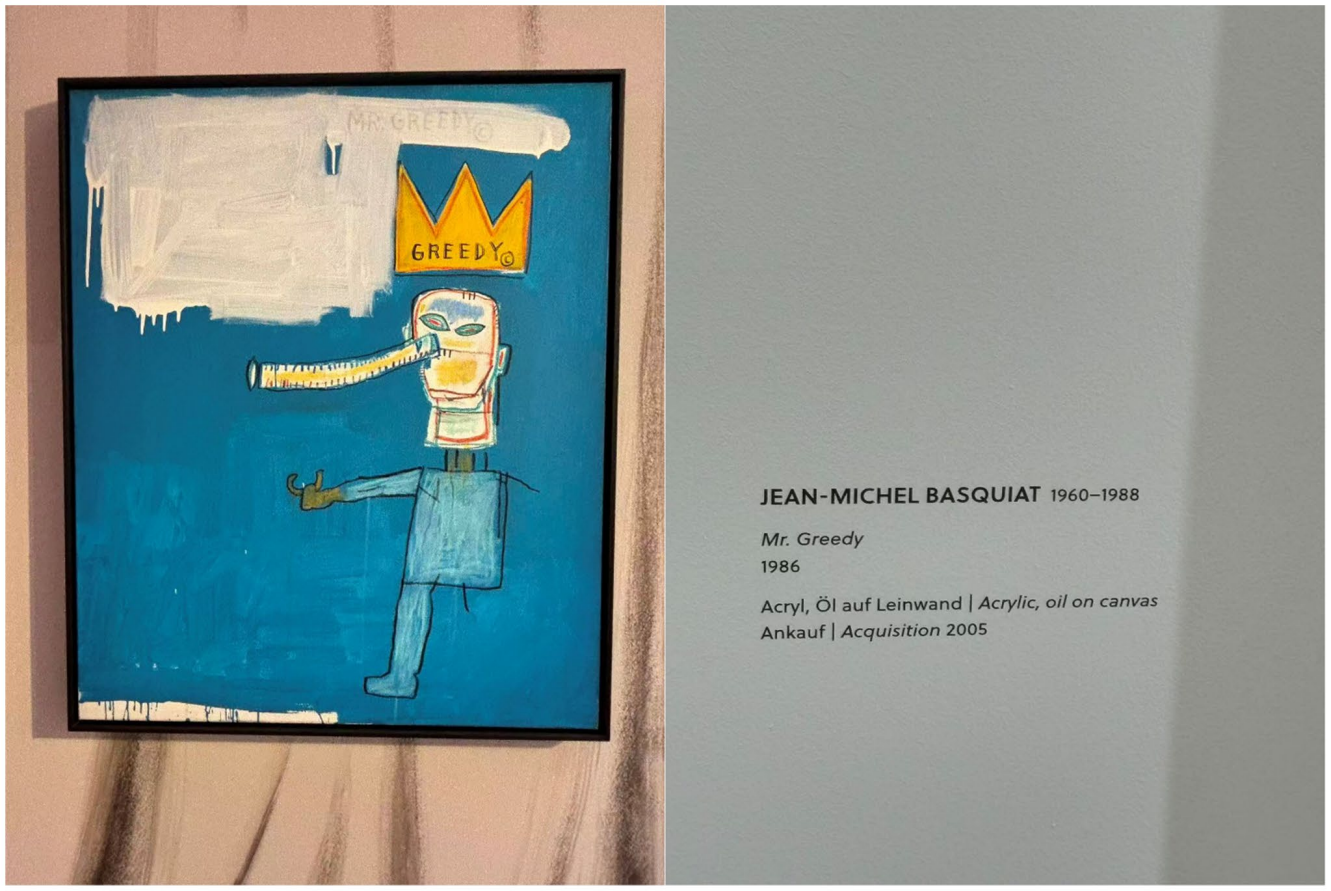
Example stimulus from the exhibition: Jean‑Michel Basquiat, Mr. Greedy (1986). Acrylic and oil on canvas. Photograph by the researcher; reproduced with permission. Courtesy of the Heidi Horten Collection. © Estate of Jean‑Michel Basquiat / Bildrecht, Vienna.

Study sessions were conducted on the ground floor of the Heidi Horten Collection (https://hortencollection.com/en/exhibitions/permanent-artistic-interventions#main). Approximately 30 paintings were presented as a coherent installation, representing a wide range of movements and artists (e.g., Vienna 1900, German Expressionism, René Magritte’s Surrealism, European and American postwar abstraction, Francis Bacon, Pop Art, ZERO, and contemporary works). This setting fit our “low narrative” concept for two reasons: (1) many works are modern (e.g., Vienna Secession, Pop Art, Surrealism), emphasizing formal features (color, line, composition) rather than elaborate storytelling; and (2) individual paintings were displayed without explanatory text or narrative labels (see Figure 2 for an example). Visitors saw only standard identifying metadata (artist, title, date, medium). This low-narrative presentation was intentional, creating a conservative test of whether viewers without specialist training could derive meaning from visual features alone and experience being moved. The paintings were chosen via #ARTfluence, a public vote determining which artworks to display. While some works may be challenging for lay viewers and include darker color palettes, they do not depict violence, blood, or other potentially distressing content.

#### 3.1.4 Questionnaire and procedure

The protocol comprised a brief pre-questionnaire, unguided free exploration of the exhibition (~20 min), and a post-questionnaire administered on-site. The pre-questionnaire assessed self-report data on art-related traits—including art interest, engagement frequency, and art knowledge assessed with the short VIVAK (Specker et al., 2020)—as well as empathy measured by the Empathic Concern subscale of the Interpersonal Reactivity Index (IRI; Davis, 1983) and Positive and Negative Affect rated on 0–10 Likert scales. The post-questionnaire probed a broad set of constructs tailored to capture the phenomenology of being moved in a painting-viewing context: self-reported being-moved intensity, arousal, discrete affective components (e.g., happiness, sadness), moving-related bodily sensations (e.g., warm feeling in the chest, moist eyes, lump in the throat, goosebumps), moving art features (color, composition, content/meaning, symbolism, texture), cognitive engagement and understanding (interest, perceived insight, re-examination), hypothesized elicitors (dearness, attachment, moral value, communal sharing), and prosocial tendencies (desire to tell someone, hug, do something kind, increased relationship commitment). Items used Likert scales (1–10 for many ratings) and binary checkboxes for art features and bodily sensations. Several items and subscales were adapted from established measures (e.g., KAMMUS II, Zickfeld et al., 2019b) and from the *Geneva Appraisal Questionnaire* and being-moved literature; details about items are provided in the Supplementary material.

After receiving task instructions and information about data protection and withdrawal rights, participants completed the pre-questionnaire. They then entered the exhibition and explored freely for approximately 20 min; they were instructed not to engage in discussion with others during exploration and to attend to paintings that felt moving to them. After exploration, participants completed the post-questionnaire, in which they reported their five most-moving paintings and provided ratings for each nominated painting (emotional intensity, embodied responses, elicitors, perceived meaning, and prosocial inclinations). All questionnaires were completed on-site via tablets or mobile phones using *QuestionPro*.

### 3.2 Results and discussion

#### 3.2.1 Emotional intensity and eliciting events

The distribution of being moved ratings differed markedly between the moving-memories and moving-paintings tasks (see Figure 1B–C and Supplementary Table 5). In the memory condition, ratings were heavily skewed toward the high end: 32.6% of responses received the maximum score of 10, and over 52% were rated 9 or above. Scores below 5 were rare (<7%), indicating that autobiographical memories reliably elicited intense emotional responses—as found in Study 1. In contrast, ratings in the painting condition were more broadly distributed. Scores ranged from 1 to 8 (full range 0–10), with the most frequent being ratings 7 (16.5%), 6 (13.5%), and 8 (13.0%). Only 1.7% of participants gave the maximum score of 10, while low ratings (1–3) occurred more frequently (sum = 30.4%), suggesting greater variability and generally lower emotional intensity of being moved by paintings.

Autobiographical memories encompassed a wide range of event types (see Table 1). Relationship-related events (39.1%) were most common, followed by art-related (23.9%), critical life events (19.6%), and nature-related experiences (17.4%). Unlike the purely visual stimuli in the painting task, these moving memories often involved complex, multimodal, and socially meaningful experiences such as friendship (6 of 46; 33.3%), separation (5 of 46; 27.8%), and funerals (33.3%). Although, in the collection context for the questionnaire, reports of art-related memory increased to 11 of 46, and live performances were also frequent (27.3%). Purely visual artworks such as photos and paintings were reported, but only once each out of 46. Compared to Study 1, participants still reported mostly relationship-related memories but at a slightly lower proportion (Study 1: 50.0%). Art-related memory had a higher proportion than critical life (Study 1: 29.5 and 15.9% for critical life and art-related, respectively). Interestingly, 7 of 46 participants reported nature-related memories, which did not appear in Study 1.

Average being moved ratings were significantly higher for memory events (median = 8.0) than for paintings (median = 4.9). Relationship and art-related memories elicited particularly intense responses (medians = 8–9), often accompanied by emotional (e.g., joy, sadness) and bodily reactions such as warmth in the chest (63.0%), moist eyes (39.1%), and goosebumps (30.4%). Event-type differences were also evident. Relationship-related memories were marked by emotional warmth and prosocial appraisals, including high ratings of closeness (median = 6.4), attachment (medians = 6.0–6.4), and intentions to connect (e.g., wanting to hug someone: median = 6.5). In contrast, critical life events were associated with more sadness (median = 5) and intense bodily reactions such as feeling “choked up” (44%) or experiencing “a lump in the throat” (78%). Art-related events, though fewer in number, still elicited strong being moved ratings (median = 9), along with high frequencies of smiling (36.4%), goosebumps (54.5%), and memory elaboration (e.g., “It made me think of someone cherished”: median = 6.0).

In summary, autobiographical events triggered more intense, diverse, and socially grounded emotional and bodily responses than visual artworks. Being moved by paintings did include a small percentage of strongly moving experiences, but such cases were relatively infrequent. The next step is to test whether the being moved by paintings reported in this study reflects the emotion as defined in affective research, or whether participants understood/used the term differently. Compared to Study 1, it appears that the context of filling out the questionnaire (in an art-collection setting) influenced participants’ reports of moving memories: art-related experiences were more often reported, and nature-related experiences also appeared to a certain degree. Participant groups also differed in many dimensions (Study 1 included people from the general community; Study 2 primarily involved Master’s students), which could be the main reason—or a combined reason—for these differences. These findings highlight the rich, multimodal, and relational contexts that underpin deeply moving life experiences. Nevertheless, paintings demonstrated the capacity to elicit substantial emotional impact—particularly in comparison to emotionally neutral stimuli. Compared to Study 1, participants in the present study reported a higher proportion of art-related memories—exceeding even critical life events—which may be due to the art-exhibition setting in which the questionnaire was completed. Still, relationship-related memories remained the most frequently recalled, underscoring the social properties of the emotion of being moved, while not appearing strictly necessary.

#### 3.2.2 Emotional profile and bodily responses (painting viewing)

**Figure 3:**
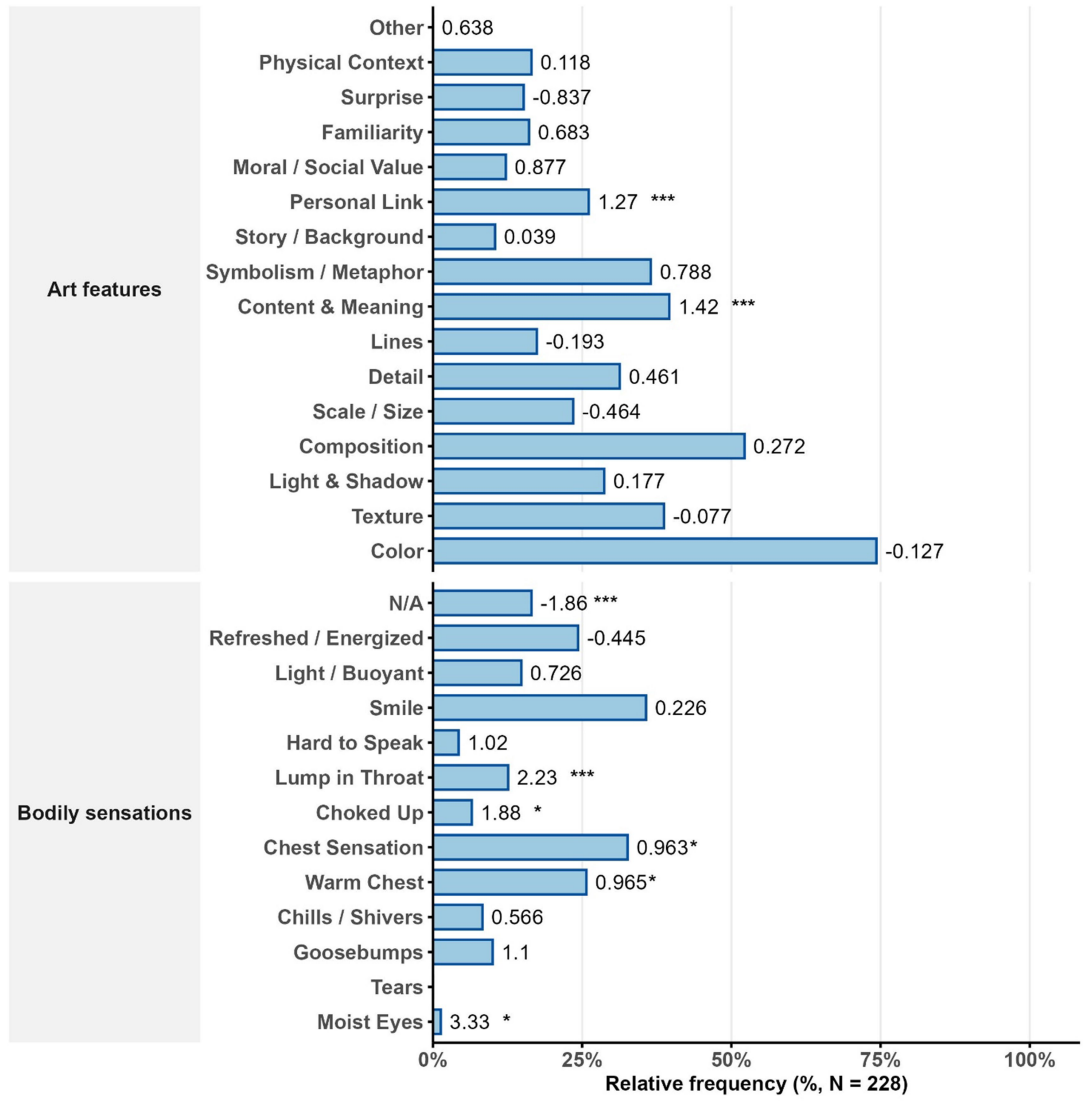
Relative frequency of reported art features and bodily sensations associated with being moved. Standardized regression coefficients (*β*) from linear mixed models correlated with being-moved ratings (right). All *p* values FDR-corrected. Significance: * *p* < 0.05, ** *p* < 0.01, *** *p* < 0.001.

As shown in Supplementary Table 6 and Figure 3, emotional ratings of self-nominated moving paintings revealed moderate levels of being moved (mean = 4.947, median = 5). Median values of being moved were significantly higher for memory events (median = 9) than for paintings (median = 5). Arousal was also moderate (mean = 4.263, median = 4), as was happiness (mean = 4.399, median = 5), whereas sadness was rated low (mean = 2.263; median = 1.5). Positive mood was rated higher than negative mood (means = 5.657 vs. 2.44).

A linear mixed model (LMM) was conducted in which continuous ratings other than being moved were entered as predictors in separate models using its rank-transformed values. Participants were included as random intercepts to account for individual differences and repeated measures. Shapiro–Wilk tests indicated that residuals showed approximate normality, with only mild deviations in a few models. Because LMMs are robust to such violations, particularly when predictors are rank-transformed, we proceeded with the reported analyses. Analyses showed that sadness (*β* = 0.326, 95% CI [0.204, 0.447], SE = 0.062, *p* < 0.001, FDR-corrected) and negative mood (*β* = 0.253, 95% CI [0.121, 0.385], SE = 0.067, *p* < 0.001) were the strongest predictors of being moved. Arousal was also associated (*β* = 0.136, 95% CI [0.005, 0.267], SE = 0.067, *p* = 0.050), although not as strongly as the previous two predictors; this differs from Cabbai et al. (2024), who reported a 95% confidence interval for the correlation between arousal and being moved of 0.69. Positive mood and happiness were not significant predictors.

**Figure 4:**
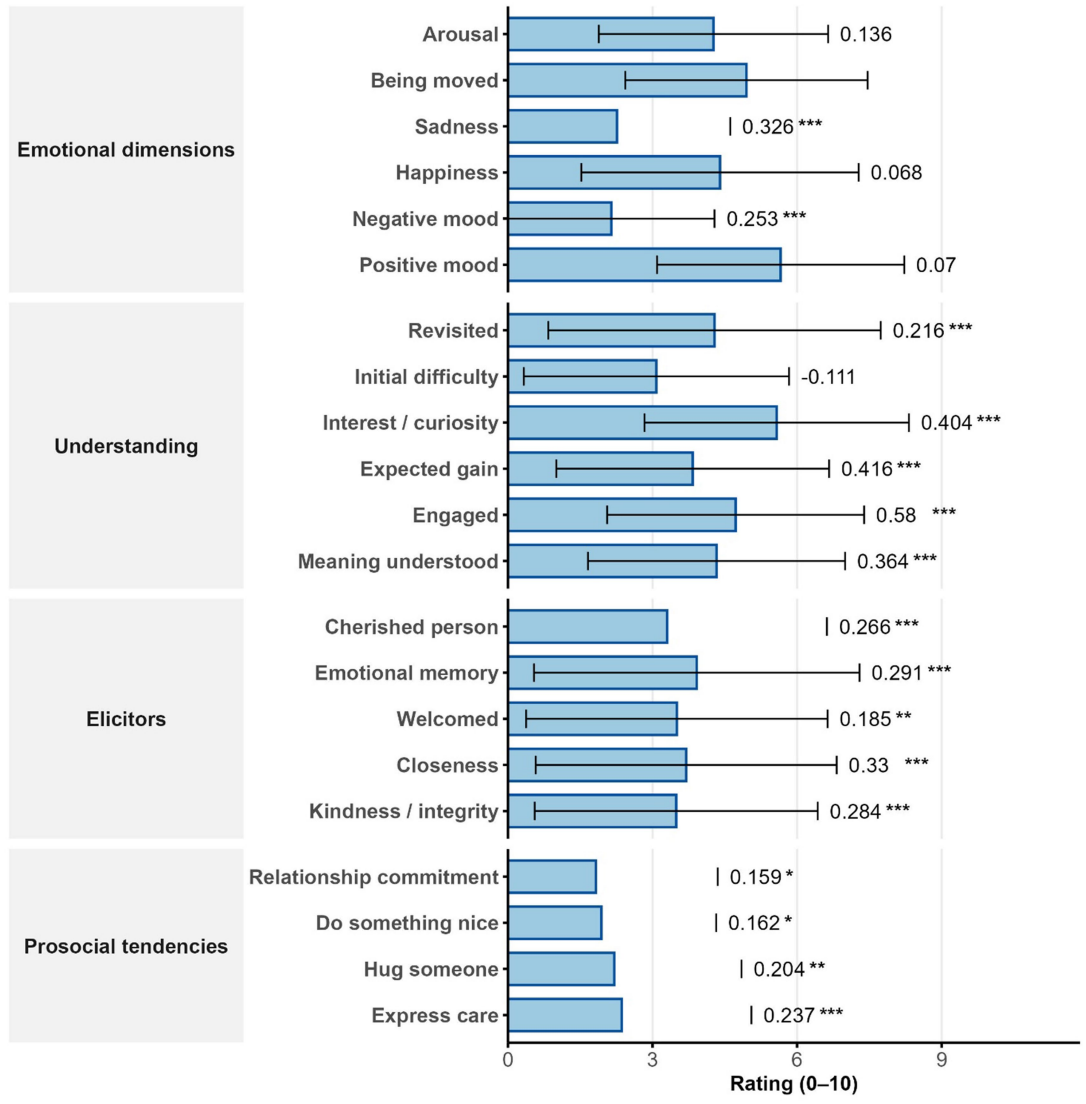
Mean ratings (0–10) of emotional dimensions, understanding, elicitors, and prosocial tendencies during the painting-viewing experience. Standardized coefficients (β) from linear mixed models predicting being-moved ratings (right). All *p* values FDR-corrected. Significance: * *p* < 0.05, ** *p* < 0.01, *** *p* < 0.001.

As shown in Supplementary Table 7 and Figures 3, 4. Participants also reported a variety of bodily responses commonly associated with being moved when viewing the paintings that affected them (see Supplementary Table 6). The most frequently reported reactions were “I smiled” (36.0%), “some feeling in the center of the chest” (32.9%), and “a warm feeling in the chest” (25.9%), followed by “refreshed or energized” (24.6%) and “goosebumps” (10.1%). Classic tear-related responses (e.g., moist eyes or crying) were absent. An LMM tested the relationships between binary bodily-response variables and being moved, with participants as random intercepts and FDR correction applied to *p* values. Residuals showed approximate normality (Shapiro–Wilk ps ≈ 0.02–0.13), and LMMs are robust to these mild deviations. Aside from the NA category (ineffective), significant predictors included a lump in the throat (*β* = 2.231, 95% CI [1.299, 3.163], SE = 0.476, *p* < 0.001), feeling choked up (*β* = 1.881, 95% CI [0.646, 3.115], SE = 0.63, *p* = 0.018), a warm feeling in the chest (*β* = 0.965, 95% CI [0.272, 1.658], SE = 0.353, *p* = 0.027), and some feeling in the chest (*β* = 0.963, 95% CI [0.276, 1.651], SE = 0.351, *p* = 0.027).

Taken together, both the emotional profile and bodily-sensation results suggest that bodily expressions of being moved in response to paintings were predominantly subtle, warm, and positively toned rather than intense or tearful. Overall, being moved by visual art was generally experienced as a warm, positive state with limited overt negative expression; however, the experience was significantly linked to sadness and arousal (rather than purely positive affect), as indicated by significant relationships with sadness, negative mood, and negatively valenced bodily sensations (e.g., a lump in the throat and feeling choked up, which showed the highest estimates). This profile also distinguishes being moved from awe, another common aesthetic emotion. Awe is typically characterized as a blend of fear and admiration (Arcangeli et al., 2020) and is associated with high arousal (Rudd et al., 2018), whereas the present findings suggest a lower-arousal, warmer pattern more consistent with being moved: warm feelings in the chest, subtle bodily tension, and elicitors related to attachment or welcome. This suggests that—even if relatively rare in painting-viewing—being moved by paintings can reflect the “literal” emotion of being moved as explored in affective research (e.g., via autobiographical memory paradigms).

#### 3.2.3 Elicitors and prosocial tendencies (painting viewing)

As shown in Supplementary Table 6 and Figure 3, participants’ perceptions of hypothesized elicitors when engaging with paintings showed moderate endorsement of morally and relationally salient content. On average, the highest ratings were given to items reflecting emotional attachment and social closeness, such as “It made me think of people or things I’m emotionally attached to” (mean = 3.917) and “I felt an exceptional sense of closeness” (mean = 3.697). Feelings of welcome were also relatively high (mean = 3.504). Perceptions of moral goodness (e.g., “The artwork conveyed kindness or moral integrity”) were rated at a similar level (mean = 3.491), while being cherished and feeling welcomed were slightly lower (means ≈ 3.27–3.303).

All five elicitor items significantly predicted being moved (LMM with FDR correction), with *p* values < 0.001 except for feeling welcomed (*β* = 0.185, 95% CI [0.06, 0.31], SE = 0.064, *p* = 0.006; passed FDR but not Bonferroni). The strongest effects were observed for perceived closeness (*β* = 0.330, 95% CI [0.206, 0.454], SE = 0.063, *p* < 0.001), emotional attachment (*β* = 0.291, 95% CI [0.17, 0.411], SE = 0.061, *p* < 0.001), and moral integrity (*β* = 0.284, 95% CI [0.163, 0.404], SE = 0.062, *p* < 0.001). All effects remained significant after FDR correction; all but feeling welcomed also passed Bonferroni correction.

Prosocial tendencies were rated more modestly overall (means ≈ 1.825–2.360; medians ≈ 1.4). Nevertheless, all four prosocial items significantly predicted being moved (FDR-corrected). The strongest associations were found for the feeling of wanting to tell someone how much one cares (*β* = 0.237, 95% CI [0.101, 0.374], SE = 0.07, *p* = 0.001), followed by wanting to hug someone (*β* = 0.204, 95% CI [0.069, 0.339], SE = 0.069, *p* = 0.005). Even subtler inclinations—such as wanting to do something kind (*β* = 0.162, 95% CI [0.018, 0.305], SE = 0.073, *p* < 0.05) or feeling more committed to a relationship (*β* = 0.159, 95% CI [0.018, 0.3], SE = 0.072, *p* < 0.05)—were positively associated with being moved.

In line with the emotional-profile and bodily-sensation findings, although median values for perceived moral/relational meaning and prosocial tendencies were low, the significant associations indicate that viewers can construct the literal emotion of being moved through painting encounters. The low descriptive levels likely reflect the overall intensity of being moved in this sample, which was moderate to low and broadly distributed. In other words, participants do not always have a moving emotion when viewing paintings, but they can sometimes construct the literal emotion of being moved by viewing low-narrative paintings—i.e., not only a thrill or intensified affect, but the being moved discussed in affective research. Thus, the lower median ratings do not imply that moving experiences in response to paintings are generally low in moral or relational meaning; rather, they reflect limited intensity in this sample. Nonetheless, the significant correlations suggest that emotional responses to moving artworks are shaped by perceived moral and relational meaning and can elicit subtle prosocial inclinations—even in the absence of explicit social interaction.

#### 3.2.4 Art features and content understanding (painting viewing)

**Figure 5:**
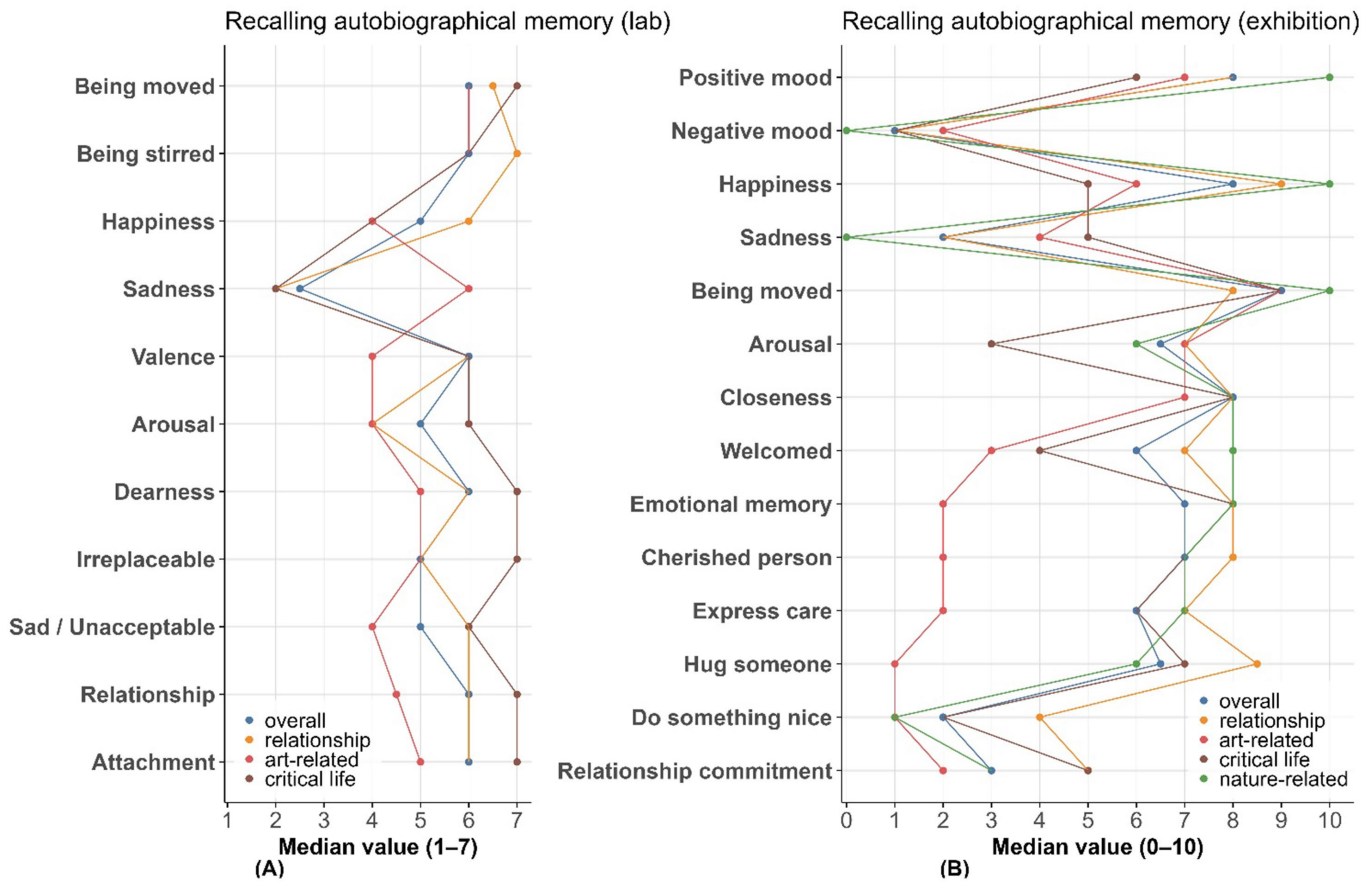
Median ratings of emotional profiles and hypothesized elicitors by event type. **(A)** Study 1: median ratings for emotional dimensions and elicitor items across moving event types (relationship, critical life, art-related, overall). **(B)** Study 2: median ratings for emotional dimensions, elicitor items, and prosocial-tendency measures across event types (relationship, critical life, art-related, nature-related, overall).

As shown in Supplementary Table 7 and Figure 4, among the visual-art features participants reported as main factors causing the moving emotion during the painting-viewing experience, the most frequently cited were color (75.30%), composition (52.26%), content or meaning (39.69%), and symbolism or metaphor (36.58%), followed by texture, detail, and personal relevance (see Supplementary Table 4). However, LMM analyses revealed that among formal and thematic predictors, only three were significantly associated with being moved after FDR correction: perceiving the artwork’s content and meaning (*β* = 1.425, 95% CI [0.813, 2.038], SE = 0.312, *p* < 0.001), its connection to one’s own experience (*β* = 1.273, 95% CI [0.58, 1.965], SE = 0.353, *p* = 0.003), and its use of symbolism or metaphor (*β* = 0.788, 95% CI [0.141, 1.434], SE = 0.33, *p* = 0.055, FDR-corrected). In contrast, basic visual properties such as color, texture, light, or scale were not significantly associated with moving responses (Figure 5).

Participants’ ratings of understanding and engagement with the artworks were comparatively high and were strong predictors of being moved. The highest mean score was for “I was interested and wanted to learn more” (mean = 5.575), followed by “I was capable of engaging meaningfully” (mean = 4.724) and “I understood the meaning” (mean = 4.329). LMM results confirmed that cognitive understanding and interest significantly predicted being moved. All variables—except initial confusion (“I found it difficult to understand at first”)—were significantly correlated with being moved under FDR correction. The strongest predictor was a sense of meaningful engagement (*β* = 0.580, 95% CI [0.468, 0.693], SE = 0.057, *p* < 0.001), followed by expected insight (*β* = 0.416, 95% CI [0.289, 0.542], SE = 0.064, *p* < 0.001) and felt interest (*β* = 0.404, 95% CI [0.277, 0.53], SE = 0.065, *p* < 0.001). Re-examining the artwork also had a positive effect (*β* = 0.216, 95% CI [0.077, 0.355], SE = 0.071, *p* = 0.004), while initial confusion was not a significant predictor.

These findings highlight the importance of thematic and interpretive features—rather than basic visual properties—in shaping being emotionally moved by visual art. While formal elements such as color and composition were frequently mentioned, only deeper, cognitively engaging aspects—such as perceived content and meaning, symbolism/metaphor, and personal relevance—emerged as significant predictors. This suggests that emotional responses to art are not merely triggered by surface-level aesthetics but rely on the viewer’s ability to extract meaning, connect the artwork to personal experience, and engage with symbolic content—consistent with the complex and multilayered nature of the emotion of being moved (Cova and Deonna, 2014).

In line with theoretical models proposed by Leder and Nadal (2014) and Pelowski et al. (2017), participants’ understanding and cognitive engagement further underscore the role of meaning-making processes in eliciting emotions like being moved. According to these models, viewers who are interested in engaging with paintings and capable of cognitive mastery are more likely to experience complex, cognitively rich emotions such as being moved. Color was likely selected most frequently because low-level features are processed early and leave a strong initial impression—hence they are often nominated—yet the correlational analyses indicate that such features are not strongly related to this complex emotion. The fact that the highest-rated item was “I was interested and wanted to learn more” suggests that curiosity, insight, and reflective engagement contribute substantially to emotional impact. The non-significant effect of initial confusion indicates that being moved is more strongly tied to successful engagement and interpretive clarity than to ambiguity or perceptual difficulty.

Taken together, these findings further support the affective nature of being moved by paintings and provide empirical perspectives emphasizing cognitive appraisal and meaning construction as central mechanisms in emotional aesthetic experience (e.g., Leder et al., 2004; Pelowski et al., 2017). The experience of being moved by visual art appears to arise less from immediate sensory impressions and more from thematic depth, cognitive mastery, and self-relevant interpretation, aligning with broader conceptions of art as a medium for emotional insight, meaning-making, and self-reflection.

#### 3.2.5 Moving memory recall: contrasts with painting viewing (moving events and art-related comparisons)

As shown in Supplementary Tables 6, 8, 9, regarding emotional profile and bodily sensations, participants reported stronger reactions in the memory condition across all measures, including positive mood (mean = 6.739 vs. 5.657), happiness (mean = 6.522 vs. 4.399), sadness (mean = 3.478 vs. 2.263), being moved (mean = 8.00 vs. 4.947), and arousal (mean = 5.674 vs. 4.263). Tear-related responses were frequent for memories (moist eyes: 39.1%; tears: 26.1%) but nearly absent for paintings (1.3, 0%). Intense expressions like feeling choked up (28.3% vs. 6.6%), a lump in the throat (34.8% vs. 12.6%), and difficulty speaking (28.3% vs. 4.4%) were also more common in memory-based experiences. Although smiling and warm or energized feelings occurred in both conditions, they were more frequent for memories (e.g., warm chest: 63.0% vs. 25.9%; refreshed: 30.4% vs. 24.6%).

Regarding perceived moral/relational meaning and prosocial tendencies, participants rated the memory condition substantially higher than the painting condition across all items. The strongest differences appeared in relational and affective elicitors, e.g., feeling an exceptional sense of closeness (median = 8 vs. 3), being welcomed (median = 6 vs. 2.5), and emotional attachment (median = 7 vs. 2). Prosocial tendencies were also markedly stronger in the memory condition, with participants more likely to feel like telling someone they care (median = 6 vs. 1), wanting to hug someone (median = 6.5 vs. 1), and even doing something extra nice (median = 2 vs. 1) or feeling more committed to a relationship (median = 3 vs. 1).

As in Study 1 (see Table 1), two raters categorized events into relationship (39.1%), critical life (19.6%), art-related (23.9%), and miscellaneous (17.4%). Interestingly, 7 of 46 events were nature-related, which did not appear in Study 1. See Supplementary Tables 8–9, Among reported moving memories, art-related events elicited emotional intensity comparable to other events, with positive mood (median = 7), being moved (median = 9), and arousal (median = 7)—slightly lower than relationship- or nature-related events. Median sadness ratings were lower than for critical life events (4 vs. 5), while negative mood was higher (2 vs. 1). Moral and relational elicitors were rated somewhat lower overall (with the exception of an exceptional sense of closeness, median = 7; particularly “thinking of someone cherished,” median = 2), and prosocial tendencies were minimal (e.g., medians: hug = 1, doing something nice to others = 1, relationship commitment = 2). Bodily responses for art-related memories showed a mixed pattern: some negatively valenced and emotionally intensified sensations were relatively frequent, including moist eyes (63.6%), goosebumps (54.5%), chills or shivers (27.3%), and regressed feelings (18.2%). Some reactions were high but still lower than for critical life events (e.g., tears: 36.4% vs. 55.6%; choked up: 36.4% vs. 44.4%), whereas warm feelings were higher than in critical life events (54.5% vs. 44.4%).

Nature-related moving events—which, like paintings, are visually salient and relatively low in social cues—showed a distinct pattern: for emotional profiles, medians for positive mood and happiness were 10 out of 10, and medians for negative mood and sadness were 0 out of 10. Arousal was moderate (median = 6). For bodily sensations, there were no tears; the strongest warm feeling (85.7%), frequent smiling (85.7%), and high rates of feeling refreshed (71.4%) were reported. Elicitor ratings were very high (e.g., welcomed, attachment, cherished), as were prosocial tendencies (e.g., medians of telling someone “I care”: nature vs. art = 7 vs. 2; and hugging someone: 6 vs. 1).

The memory condition thus evoked strong emotional and bodily responses across event types, highlighting the intensity and depth of autobiographical moving experiences and providing a meaningful comparison with being moved by paintings. Given the ceiling effect in autobiographical memory ratings—and considering differences in temporal distance, context richness, and modality—it is not appropriate to compare the two experiences (painting viewing vs. memory recall) as equivalent in all descriptive and predictive dimensions, apart from frequency and intensity. Still, differences among memory types reveal informative patterns. These memories involved both positive and negative affect and were often accompanied by embodied expressions—such as tearfulness or speech disruption—suggesting that being moved through memory is not only cognitively accessible but also physically re-experienced. They were rich in relational and moral meaning, frequently linked to closeness, attachment, and prosocial impulses (e.g., expressing care or deepening social bonds), supporting the view of being moved as a socially embedded emotion when grounded in personal significance.

Art-related memories were more mixed—combining negative mood and bodily sensations with positive ones. They were emotionally intense, with higher frequencies of goosebumps and chills and lower frequencies of feeling refreshed/energized. Social aspects—including social elicitors and prosocial tendencies—were comparatively lower than in all other types, including nature-related memories. Interestingly, nature-related memories, which were not reported in Study 1’s laboratory context, appeared here to a notable degree. They were purely positive (even if sometimes moving to tears was implied), and relaxed; yet they also showed very high ratings in relationship-related dimensions (elicitors and some prosocial tendencies). With very low narrative and no explicit social stimuli, nature-related memories merit further investigation to understand the nature of being emotionally moved.

Among memory types, both art- and nature-related events were reported but differed in emotional tone. Art-related memories were emotionally intense and aesthetically reflective, yet less relational and prosocial. In contrast, nature-related memories more often evoked awe, connection, and interpersonal warmth, illustrating how different contexts shape the emotional and social qualities of being moved—even within autobiographical recall.

### 3.3 Summary

Study 2 investigated whether participants in a museum exhibition context experienced the complex emotion of being moved as described affective research. Standardized to a 0–10 scale, participants showed moderate Empathic Concern (median 5.43, range 3.71–7.43); a generally pleasant baseline affect with Positive Affect at 7.00 (1.00–9.00) and Negative Affect at 2.00 (0.00–9.00); high Art Interest (median 7.00, 2.00–10.00); low Art Knowledge (median 3.00, 0.00–10.00); and infrequent art engagement (frequency median 1.67, range 0.00–4.44). Detailed information is provided in the Supplementary Tables 11, 12; this pattern aligns with expectations for a non-expert sample that has limited prior art knowledge but engages with artworks effectively due to high interest. Compared to autobiographical recall, painting-related moving experiences were less intense, less frequent, and more variable across individuals. Nonetheless, they retained several hallmarks of the emotion of being moved, including links to sadness, embodied sensations (warmth in the chest, lump in the throat), and modest but significant associations with prosocial tendencies.

Crucially, predictors of being moved in the painting condition were not surface-level visual features such as color or composition, but rather thematic and interpretive elements: perceived content and meaning, symbolism, and personal relevance. Cognitive engagement—interest, insight, and meaningful re-examination—emerged as particularly strong predictors. These findings highlight that meaning-making processes, rather than formal aesthetics alone, are central in generating moving experiences with visual art.

The comparison with autobiographical memories underscores the distinction between the two contexts. Memories evoked higher emotional intensity, stronger bodily reactions (including tears), and more robust prosocial inclinations, with relationship-related events dominating the reports. By contrast, paintings tended to evoke subtler, more positively toned experiences. Notably, nature-related memories—absent in Study 1—emerged as a new category, suggesting that non-social, low-narrative contexts may also elicit the emotion of being moved in ways that resemble art-related experiences.

However, the sample for Study 2 consisted primarily of Master’s psychology students—a relatively homogeneous, young, and potentially art-interested group. This may introduce bias, as their academic background and potential familiarity with psychological or artistic concepts could shape how they interpret and report moving experiences, which limits the generalizability of the findings.

Taken together, Study 2 provides evidence that participants do sometimes experience the literal emotion of being moved while viewing paintings, even in the absence of narrative scaffolding—at least among young adults with potential interest in art. However, such responses are typically less intense than autobiographical moving experiences, and they rely heavily on viewers’ ability to construct meaning, establish personal relevance, and engage cognitively with the artwork. These findings refine our understanding of art-elicited moving experiences and point to the interpretive demands and contextual factors that shape them.

## 4 Study 3

Study 2 showed that individual paintings in a low-narrative museum setting can elicit experiences of being moved, though typically of moderate intensity and closely tied to meaning-making and personal relevance. Study 3 extended this inquiry from single works to the level of a whole exhibition visit. Instead of focusing on self-nominated moving paintings, participants reported on their affective state across an entire museum experience, providing a broader perspective on how being moved manifests during naturalistic art engagement. Importantly, this study employed a non-directive design: participants completed a comprehensive emotion inventory without being explicitly primed to consider “being moved,” thereby reducing demand characteristics and offering a more ecologically valid measure of spontaneous emotional responses.

### 4.1 Methods

Thirty-two participants (median age = 24; age range 21–30; 18 female; 2 preferring not to disclose) took part in the study as volunteers recruited from a Master’s-level course in the Department of Psychology, University of Vienna. Ethical considerations are discussed alongside Study 2 in Section 3.1.1. All participants provided demographic information and self-report measures of art-related traits (art interest, engagement frequency, and art knowledge), Empathic Concern, and Positive and Negative Affect, using the same instruments as Study 2. The study followed the same ethical and procedural provisions as Study 2: participants were informed about the purpose of the research, data protection and anonymity procedures, and their right to withdraw at any time; the questionnaires were anonymous, and participation was indicated by an explicit agreement item at the start of the survey.

The measurement protocol drew on instruments used in museum-aesthetics research (notably measures and approaches developed in Pelowski’s work on museum experience; Pelowski et al., 2018; Pelowski et al., 2017; Pelowski and Akiba, (2011)) and incorporated items employed in Studies 1–2. Pre-visit measures included self-reported art knowledge, art interest, frequency of art engagement, sense of connection with others (self–other relationship), baseline empathy, and baseline prosociality. The post-visit questionnaire assessed a broad battery of emotion and feeling items referring to the whole exhibition experience, including tenderness, compassion, hope, guilt, shame, remorse, love, happiness, shock, boredom, anger, playfulness, sadness, sudden insight, anxiety, sense of beauty, pleasure, being moved, sense of transformation, desire to change oneself, thrill, and engagement. The instrument was also designed to produce difference scores for empathy, prosociality, positivity, negativity, and arousal to capture short-term changes associated with the visit; full item wording and response anchors are provided in the Supplementary materials.

On arrival, participants completed the pre-visit questionnaire onsite, then explored the Heidi Horten Collection freely for approximately 20 min with instructions not to talk to other visitors. The exhibition context matched that of Study 2: paintings were presented in a low-narrative format (standard identifying metadata such as artist, title, and year were visible, but no individual interpretive captions were provided). This low-narrative presentation provided a conservative, ecologically valid test of whether visual features and internal interpretation alone can produce being-moved experiences. Participants completed the pre- and post-visit questionnaires on tablets or smartphones via *Qualtrics*; all data were collected electronically on site.

### 4.2 Results

Standardized to a 0–10 scale, participants—consistent with Study 2—reported high art interest (median 8.0, range 1.0–10.0), low art knowledge (median 2.0, 0.0–10.0), infrequent art engagement (median 2.3, 1.0–4.0), a moderately pleasant baseline affect (Positive Affect median 6.0, 3.0–10.0; Negative Affect median 2.5, 0.0–9.0), and high Empathic Concern (median 7.5, 3.0–10.0). Detailed information is provided in the Supplementary Table 12.

As shown in Figure 1D and Supplementary Table 5, Participants’ ratings of being moved during the exhibition were modest overall and broadly distributed. The most frequent rating was 6 (18.8%), with no participants selecting the maximum values (9 or 10). Median rating was 5 (range 1–8), suggesting that intense episodes were uncommon but that moderate moving experiences did occur even without priming.

As shown in Supplementary Table 10, the overall affective tone of the visit was positive (median positive mood = 7), with moderate arousal (median = 4) and low negative mood (median = 2). On discrete emotions, participants reported high scores for happiness (median = 6.5), beauty (median = 7), pleasure (median = 6), and being moved (median = 5). Negative states (sadness, anxiety, boredom, shock, shame, guilt, remorse) were infrequent (medians = 0–1).

Considering the sample size and the non-normality of the data, we applied Spearman correlational analyses to explore how emotional components were associated with being moved. Although none survived FDR correction, the strongest associations were tenderness (*ρ* = 0.483, *p* = 0.005), self-reflection (“need to change something about myself,” ρ = 0.447, *p* = 0.010), hope (ρ = 0.407, *p* = 0.021), pleasure (ρ = 0.400, *p* = 0.023), and compassion (ρ = 0.399, *p* = 0.024). These patterns suggest that, in this context, being moved co-occurred with gentle, prosocial, and self-reflective states rather than high-arousal sadness.

### 4.3 Discussion

Study 3 explored the affective profile of a whole museum visit in a low-narrative context. Participants’ reports showed that experiences of being moved did occur but were modest in intensity, closely mirroring the distribution observed for single-painting ratings in Study 2. The visit as a whole was characterized by positive affect, beauty, and pleasure, with only minimal negative affect.

Associations between being moved and tenderness, compassion, hope, pleasure, and self-reflective transformation suggest that moving experiences in this context are less about intense sadness and more about contemplative, prosocial, and beauty-inflected states. Although these correlations did not reach corrected significance, the pattern points to consistent tendencies that align with theoretical accounts of being moved as a mixed but predominantly positive social-moral emotion.

A notable strength of Study 3 was its non-directive design: participants were not primed to think about being moved but instead completed a broad emotion inventory. This reduces expectancy effects and increases ecological validity, capturing spontaneous affective responses rather than task-induced reports. The findings therefore provide a conservative estimate of being moved in naturalistic painting-viewing conditions.

However, as in Study 2, the sample consisted primarily of Master’s psychology students, a relatively homogeneous group. This demographic may have influenced the findings, as these participants are likely to have some interest in art and familiarity with psychological concepts, which could shape their responses. The limited diversity of the sample makes it difficult to generalize the results to broader populations.

Taken together, Study 3 suggests that people in low-narrative museum visits can feel being moved, the same at least for psychological students, but typically in a subtle, reflective form tied more to beauty, compassion, and personal reflection than to intense sadness or bodily tears. This pattern complements the findings of Studies 1 and 2, highlighting both the distinctiveness and the limitations of being moved in visual art contexts compared with autobiographical or multimodal experiences.

## 5 General discussion

This paper set out to explore how likely it is for people to experience being emotionally moved in response to visual art, and what features characterize such experiences in comparison to autobiographical or multimodal contexts. Using a triangulation of methods across three studies — autobiographical memory recall (Study 1), self-nominated moving paintings and memory reports during a museum visit (Study 2), and a whole-visit assessment without priming (Study 3) — we obtained convergent insights into both the likelihood and the phenomenology of being moved in visual-art contexts, at least for a lay but art-interested population such as psychology students.

Given the small and relatively homogeneous samples (primarily Master’s students), the findings should be interpreted cautiously and not generalized without further research. Nevertheless, these studies provide an initial step toward understanding the emotion of being moved in visually dominated contexts such as painting exhibitions. The following sections synthesize these findings in relation to prior research, highlight their implications, and outline limitations and directions for future work.

### 5.1 How likely are people to feel moved when viewing visual artworks?

Across studies, we found a consistent pattern. Autobiographical recall tasks (Studies 1 and 2) reliably produced intensely moving reports, confirming the effectiveness and robustness of this method widely used in affective research. In contrast, art-museum experiences — whether based on self-nominated paintings or whole-visit reports without priming — showed ratings broadly distributed across the scale, with only a small proportion of very high scores. Intense “peak” experiences of being moved were thus relatively rare in the painting-viewing context.

Autobiographical memories tied to relationships were most likely to elicit strong moving experiences, but non-social events — including some critical life experiences, art-related encounters, and nature-related experiences — could also be deeply moving. In contrast, encounters with low-narrative paintings typically produced moderate reports of being moved, with strong responses occurring in a minority of cases. Taken together, these findings suggest that narrative richness, social relevance, and semantic content amplify the intensity and frequency of being moved, while purely visual stimuli — even in the absence of narrative scaffolding — retain the capacity to evoke the emotion in a measurable way.

The results also indicate that being moved is not reducible to arousal. While arousal showed some associations with being moved, sadness and negative mood were more consistent predictors. This contrasts with earlier aesthetic research emphasizing arousal in responses to visual art (e.g., Vessel et al., 2012) or reporting stronger arousal–being moved correlations (Cabbai et al., 2024). Our findings therefore highlight that being moved in visual-art contexts may not overlap fully with the “aesthetic thrill” or arousal-dominated responses often studied in empirical aesthetics.

### 5.2 What are the characteristic features of being moved in the visual-art context?

When participants nominated moving paintings, reported elicitors and prosocial tendencies were generally low in absolute levels. However, correlations told a different story: all hypothesized elicitors (moral goodness, closeness, welcome, attachment, cherishment) and all prosocial tendencies (wanting to tell someone they care, hug, help, or commit more strongly to a relationship) significantly predicted being moved. This aligns with the Kama Muta Multiplex Scale (Zickfeld et al., 2019b), which integrates such relational and prosocial appraisals as central predictors of being moved.

Besides arousal, sadness emerged as a stronger predictor than general negative mood, consistent with prior work linking sadness and being moved in film (Hanich et al., 2014; Wassiliwizky et al., 2015) and music (Vuoskoski and Eerola, 2017). Predictive bodily sensations — moist eyes, lump in the throat, feeling choked up — further support the presence of sadness within the being-moved response to paintings.

Despite limited art knowledge and the absence of explanatory labels, participants reported high interest, meaningful engagement, and perceived understanding. Being moved correlated strongly with perceived meaning, personal relevance, re-examination, and insight. Predictive features included content and meaning, personal linkage, and symbolism/metaphor. This suggests that viewers actively construct understanding based on their own experiences, filling in narrative gaps and forming bonds between artwork and self. Such findings align with constructivist emotion theories (Barrett and Russell, 2014; Barrett, 2017) and with aesthetic appreciation models (Leder et al., 2004; Leder and Nadal, 2014; Pelowski et al., 2017), which emphasize cognitive mastery and self-related interpretation as key processes leading to complex emotions like being moved.

When asked to report on an entire museum visit without priming the term “being moved,” participants again showed a broadly distributed but modest pattern of ratings. Interestingly, being moved here correlated not with sadness but with pleasure, tenderness, compassion, hope, and self-reflection. Although limited by sample size, this suggests that being moved in visual-art contexts can also take a predominantly positive, reflective form — consistent with accounts of “tears to transformation” (Pelowski, 2015) and with recent findings highlighting tenderness (Campeggiani, 2021; Cullhed, 2024), compassion (Deonna, 2018; Zickfeld et al., 2019a), and hope as facets of being moved (Menninghaus et al., 2015).

Together, these results show that purely visual art can sometimes elicit genuine emotion of being moved, but often through cognitive construction and self-relevance rather than through intense sadness or narrative-driven cues.

### 5.3 How does visual art-elicited being moved diverge from being moved in multimodal or autobiographical contexts?

The autobiographical memory condition exhibited a pronounced ceiling effect: ratings were strongly skewed toward the top of the scale, compressing variance and attenuating correlations. This restricted range undermines straightforward correlational comparisons and makes it problematic to treat memory reports as an unbiased baseline for evaluating predictors of in-situ painting responses. Beyond these statistical issues, autobiographical and in-situ painting experiences differ fundamentally in elapsed time since the event, contextual richness, modality, and degree of personal involvement — factors that complicate direct comparison.

Nevertheless, categorical analyses of art-related memories, together with relationship- and life-event-related memories, remain informative. Compared with relationship-related experiences, art-related memories tended to be more inwardly focused, received the lowest median ratings for socially oriented elicitors and prosocial tendencies, and the highest median ratings for sadness and negative mood in both Studies 1 and 2. Prior work has shown that art content can eliminate avoidance tendencies toward negative emotions (Wagner et al., 2014; Menninghaus et al., 2019), making individuals more likely to accept and even enjoy negative feelings, especially sadness, in art appreciation (Menninghaus et al., 2017). This explains why being moved by art is often characterized by elevated negative mood, while still being strongly mixed with positive emotions.

Interestingly, nature-related moving experiences, which appeared in a notable proportion of autobiographical memories collected in the exhibition context, were reported as evidently and purely positive. They displayed the highest levels of elicitor activation and the second-highest levels of prosocial tendencies. Unlike art-related or relationship-related events, these experiences occurred without social interaction and depended primarily on visual perception, yet still produced a distinct emotional profile. Caracciolo (2021) has argued that being moved by nature is constructed in ways that differ from, or complicate, the ecological sublime by emphasizing human societies’ moral and material entanglement with nonhuman realities. To deepen our understanding of the emotion of being moved, it seems essential to study moving experiences with nature alongside those with art.

It is important to emphasize that lower mean prosocial or moral ratings in art contexts do not imply that social appraisals are irrelevant. Even modest endorsements of dearness, attachment, or perceived moral value significantly predicted being-moved intensity, suggesting that social–affective mechanisms, although attenuated, remain operative in responses to visual art.

### 5.4 Limitations and future directions

Although this research provides an ecologically grounded initial characterization of how low-narrative paintings can elicit experiences of being moved, limitations constrain the strength and scope of the conclusions. First, as discussed, the studies were exploratory and statistically underpowered for some inferential questions: sample sizes were modest, and participants were primarily convenience samples (mainly Master’s students and their acquaintances). These factors might reduce generalizability and increase the risk of false positives. Second, measurement relied almost exclusively on self-report questionnaires and checklist items; linguistic ambiguities (e.g., subtle differences between the German terms *bewegt* and *gerührt*) and conceptual overlap between social–affective being moved and aesthetic-judgment intensity limit interpretability. Third, the field design traded experimental control for ecological validity: participants varied in baseline mood, viewing duration, familiarity with artworks, and situational factors such as crowding or time of day. While individual differences were statistically controlled in Study 2, they were not addressed in the other studies due to small sample sizes. Moreover, all data were drawn from a single permanent exhibition in Vienna, constraining cultural and contextual generalization. Finally, analytic challenges remain: multiple correlations were tested (some did not survive correction), event coding would benefit from reported inter-rater reliability, and future studies could employ mixed-effects models, mediation or moderation analyses, and robust multiple-comparison procedures to more precisely isolate underlying mechanisms.

However, the combined studies provide a first, important step. Yet, to address the above issues, future research should prioritize preregistered, adequately powered replications with more diverse samples and multi-method approaches; experimentally manipulate key variables (e.g., narrative framing, presence or absence of explanatory text, multimodal augmentation, time-on-task, and social presence); incorporate physiological and behavioral measures alongside self-report and qualitative interviews; improve and report the reliability of coding schemes; and apply robust statistical methods. Cross-cultural replications and comparisons across exhibition types (narrative vs. low-narrative, single-work vs. immersive installations) will further clarify the boundaries of these findings.

In summary, this series of exploratory studies demonstrates that even in low-narrative exhibition contexts, purely visual artworks can — through interpretive engagement and personal relevance — elicit experiences that participants describe as being moved. Compared with autobiographical and multimodal stimuli, painting-elicited being moved differs in intensity, bodily expression, and social–affective profile: it tends to be more inwardly focused, interpretive, and subtly embodied, often embedded within a broadly positive aesthetic experience. However, more research is needed to test causal mechanisms and to clarify the specific role of the visual modality within the broader emotional repertoire of being moved.

## 6 Conclusion

Even in low-narrative exhibition settings, viewers of purely visual artworks can experience the distinct emotion labeled as being moved—at least among lay people with some interest in art, uch as the psychology students in our sample. Compared to autobiographical or multimodal events, however, painting-elicited being moved tends to be less intense, less socially embedded, and more inwardly reflective. It often arises through meaning-making, personal relevance, and cognitive mastery, manifesting as a subtle, beauty-inflected state tied to tenderness, compassion, or hope rather than to overwhelming sadness. These findings underscore the importance of considering modality, context, and interpretive engagement when theorizing about being moved, and call for further empirical work to map the distinct but overlapping pathways through which art and life evoke this complex and multifaceted emotion.

## Data Availability

The raw data supporting the conclusions of this article will be made available by the authors, without undue reservation.

## Footnotes

1 We adopt a constructionist view of emotion (e.g., Gross and Feldman Barrett, 2011; Barrett, 2017). We retain terms such as “elicit” and “stimulus” for readability, but use them to denote person–artifact interactions that afford meaning-making. In this paper, an “elicitor” refers to an interactional configuration in which attachment, communal sharing, or appraisals of irreplaceability are activated, thereby increasing the likelihood of feeling moved; it does not imply simple stimulus-to-response causation by artwork features alone.

2 Vessel et al. (2012, 2019) tested “being aesthetically moved” via the question “how strongly does this painting move you?” according to the following instructions: “Imagine that the images you see are of paintings that may be acquired by a museum of fine art. The curator needs to know which paintings are the most aesthetically pleasing based on how strongly you as an individual respond to them. Your job is to give your gut-level response, based on how much you find the painting beautiful, compelling, or powerful. Note: The paintings may cover the entire range from ‘beautiful’ to ‘strange’ or even ‘ugly.’ Respond on the basis of how much this image ‘moves’ you. What is most important is for you to indicate what works you find powerful, pleasing, or profound.”
