# Supplementary material for: Being moved by modern abstract art
Source: Front Psychol. 2026 Feb 13;17:1720357. doi: 10.3389/fpsyg.2026.1720357 (PMC12946045; doi:10.3389/fpsyg.2026.1720357)
Supplement: Supplementary file 1 [file Table_1.docx]

1. **Questionnaires and Key Items**

**Elicitors assessed in Study 1 (English Translation).** Participants were asked to reflect on the primary factor that most strongly evoked their feelings of being warmly moved and the tendency or presence of tears. This factor was referred to as “X1.”

They were instructed to briefly describe X1 (in a few words or one sentence) and to rate their agreement with the following statements on a 7-point Likert scale (1 = not at all, 7 = extremely):

1. I perceive X1 as very dear to me (Dearness).
2. X1 is radically irreplaceable for me (Irreplaceable).
3. I feel extremely sad and find it unacceptable when I imagine being without X1 (Sad/Unacceptable).
4. I feel a stronger relationship with X1 during and/or after experiencing this event (Relationship).
5. I feel attached to X1 (Attachment).

**Scales assessed in Study 2.** Binary variables included art features and bodily sensations. For art features, participants were asked: “What are you most emotionally moved by in this artwork? Please select the main factor(s). You may choose more than one if applicable.” The available options were: Color, Texture, Light and shadow, Composition, Scale (size), Detail, The use of lines, Content & Meaning, Symbolism / Metaphor, Background story, Link to my own experience, Moral or social values, Familiarity, Surprise, Physical context (e.g., lighting, spatial arrangement), Others.

For bodily sensations, participants were asked: “While viewing this artwork, you felt/experienced… Please select all options that apply.” The available options were: Moist eyes, Tears, Goosebumps or hair standing up, Chills or shivers, A warm feeling in the center of the chest, Some feeling in the center of the chest, Choked up, A lump in the throat, Difficulty speaking, I smiled, Buoyant or light, Refreshed/energized/exhilarated, N/A.

Emotional dimensions, understanding, elicitors, and prosocial tendencies were assessed using a 0–10 Likert scale. For emotional dimensions (0 = not at all, 10 = very much), participants responded to the question: “While viewing this artwork or recalling this memory, you felt/experienced…” The items included Positive mood, Negative mood, Happiness, Sadness, Being moved, Arousal (e.g., feeling active or energized).

Understanding was assessed (0 = not at all, 10 = completely true) with the instruction: “While viewing this artwork, please indicate to what extent each of the following statements describes your actions.” The items were:

1. I understood the meaning or message of the artwork. (Meaning understand)
2. I was capable of engaging meaningfully with the artwork. (Engaged)
3. I expected to gain something from viewing the artwork. (Expected gain)
4. I was interested in the artwork and wanted to learn more about it. (Interest/curiosity)
5. I found the artwork somewhat difficult to understand at first. (Initial difficulty)
6. I revisited the artwork at least once to help myself better understand it. (Revisited)

Elicitors were rated (0 = not at all, 10 = completely true) in response to the question: “While viewing this artwork, please indicate to what extent each of the following statements describes your experience.” The items were:

1. I felt that the artwork conveyed kindness or moral integrity. (Kindness/integrity)
2. I felt an exceptional sense of closeness (with someone or something). (Closeness)
3. I felt an extraordinary feeling of welcoming or being welcomed. (Welcomed)
4. It made me think of people or things I’m emotionally attached to. (Emotional memory)
5. It made me think of someone or something that felt deeply cherished. (Cherished person)

Prosocial tendency were assessed (0 = not at all, 10 = completely true) using the question: “After viewing this artwork, please indicate to what extent each of the following statements describes your feelings.” The items were:

1. I felt like telling someone how much I care about them. (Express care)
2. I wanted to hug someone. (Hug someone)
3. I wanted to do something extra nice for someone. (Do something nice)
4. I felt more strongly committed to a relationship. (Relationship commitment)

**Emotions and feelings assessed in Study 3.** Emotions and feeling were assessed in two blocks, items were rated on a 0–10 scale (0 = not at all, 10 = very much so). In Block 1, question: “While I was viewing the artworks, I felt/experienced…” The listed emotions included Love, Happiness, Being shocked, Boredom, Anger, Playfulness, Sadness, (Sudden) insight, Anxiety, A sense of beauty, Pleasure, Being moved, A transformation, Changing (something about) myself, Being thrilled, A sense of active engagement.

In Block 2, participants responded to the same question and rated the extent of which they experienced the following emotions: Tenderness, Compassion, Hope, Guilt, Shame, Remorse.

1. **Supplementary Tables**

Supplementary Table 1. Frequency distribution of “being moved” ratings by context (*Bewegt* vs. *Gerührt*, group vs. alone; Study 1)

| **Scale / context** | | **N** | **Rating** | | | | | | |
| --- | --- | --- | --- | --- | --- | --- | --- | --- | --- |
|  |  |  | **1** | **2** | **3** | **4** | **5** | **6** | **7** |
| *Bewegt* (group) | n | 22 | 0 | 1 | 1 | 0 | 3 | 5 | 12 |
|  | % | - | 0 | 4.5 | 4.5 | 0 | 13.6 | 22.7 | 54.5 |
| *Gerührt* (group) | n | 22 | 1 | 0 | 1 | 0 | 4 | 4 | 12 |
|  | % | - | 4.5 | 0 | 4.5 | 0 | 18.2 | 18.2 | 54.5 |
| *Bewegt* (alone) | n | 22 | 0 | 0 | 1 | 0 | 1 | 11 | 9 |
|  | % | - | 0 | 0 | 4.5 | 0 | 4.5 | 50 | 40.9 |
| *Gerührt* (alone) | n | 22 | 0 | 0 | 1 | 1 | 4 | 8 | 8 |
|  | % | - | 0 | 0 | 4.5 | 4.5 | 18.2 | 36.4 | 36.4 |
| Overall | n | 88 | 1 | 1 | 4 | 1 | 12 | 28 | 41 |
|  | % | - | 1.1 | 1.1 | 4.5 | 1.1 | 13.6 | 31.8 | 46.6 |

Supplementary Table 2. Ratings and correlations with “being moved” (combined across contexts; Study 1)

| **Scale** | **Median** | **IQR** | ***Bewegt*** | | ***Gerührt*** | |
| --- | --- | --- | --- | --- | --- | --- |
|  |  |  | **r** | **p(FDR)** | **r** | **p(FDR)** |
| *Bewegt* | 6 | 1 | - | - | 0.677 | 0.000 |
| *Gerührt* | 6 | 2 | 0.677 | 0.000 | - | - |
| Happiness | 5 | 4.25 | 0.123 | 0.510 | 0.290 | 0.132 |
| Sadness | 2.5 | 4.25 | 0.091 | 0.589 | -0.102 | 0.576 |
| Valence | 6 | 3 | 0.135 | 0.507 | 0.252 | 0.163 |
| Arousal | 5 | 3 | 0.531 | 0.004 | 0.305 | 0.132 |
| Dearness | 6 | 2 | 0.305 | 0.132 | 0.355 | 0.127 |
| Irreplaceable | 5 | 3 | 0.269 | 0.163 | 0.362 | 0.127 |
| Sad / Unacceptable | 5 | 2.25 | 0.056 | 0.729 | 0.138 | 0.507 |
| Relationship | 6 | 3 | 0.260 | 0.166 | 0.306 | 0.132 |
| Attachment | 6 | 2 | 0.274 | 0.163 | 0.311 | 0.132 |

Supplementary Table 3. Correlations of “being moved” (*Bewegt* and *Gerührt*) with scales by context (Study 1)

| **Term** | **Scale** | **Group** | | **Alone** | |
| --- | --- | --- | --- | --- | --- |
|  |  | **r** | **p(FDR)** | **r** | **p(FDR)** |
| *Bewegt* | Happiness | -0.047 | 0.886 | 0.294 | 0.302 |
|  | Sadness | 0.391 | 0.261 | -0.261 | 0.362 |
|  | Valence | -0.157 | 0.698 | 0.481 | 0.144 |
|  | Arousal | 0.504 | 0.124 | 0.602 | 0.055 |
|  | Dearness | 0.187 | 0.698 | 0.470 | 0.144 |
|  | Irreplaceable | 0.349 | 0.394 | 0.159 | 0.491 |
|  | Sad / Unacceptable | -0.159 | 0.698 | 0.192 | 0.442 |
|  | Relationship | 0.106 | 0.748 | 0.441 | 0.144 |
|  | Attachment | 0.138 | 0.748 | 0.434 | 0.144 |
| *Gerührt* | Happiness | 0.390 | 0.261 | 0.194 | 0.436 |
|  | Sadness | 0.103 | 0.748 | -0.322 | 0.269 |
|  | Valence | 0.215 | 0.698 | 0.318 | 0.269 |
|  | Arousal | 0.203 | 0.698 | 0.439 | 0.144 |
|  | Dearness | 0.528 | 0.124 | 0.230 | 0.406 |
|  | Irreplaceable | 0.513 | 0.124 | 0.208 | 0.436 |
|  | Sad / Unacceptable | -0.032 | 0.893 | 0.236 | 0.406 |
|  | Relationship | 0.229 | 0.698 | 0.384 | 0.213 |
|  | Attachment | 0.176 | 0.698 | 0.440 | 0.144 |

Supplementary Table 4. Distribution of reported emotions across moving event types (Study 1)

| **Emotion** | **Relationship** | | **Critical life** | | **Art-related** | | **Miscellaneous** | | **Overall** | |
| --- | --- | --- | --- | --- | --- | --- | --- | --- | --- | --- |
|  | **n** | **%** | **n** | **%** | **n** | **%** | **n** | **%** | **n** | **%** |
| Pleasure | 4 | 22.7 | 6 | 38.5 | 2 | 28.6 | 0 | 0.0 | 12 | 27.3 |
| Shame | 0 | 0.0 | 0 | 7.7 | 1 | 14.3 | 1 | 50.0 | 2 | 4.5 |
| Anxiety | 2 | 4.5 | 4 | 46.2 | 0 | 0.0 | 1 | 50.0 | 7 | 15.9 |
| Helplessness | 3 | 13.6 | 3 | 30.8 | 5 | 57.1 | 1 | 50.0 | 12 | 27.3 |
| Rage | 0 | 0.0 | 3 | 30.8 | 1 | 14.3 | 1 | 50.0 | 5 | 11.4 |
| Surprise | 4 | 22.7 | 3 | 15.4 | 0 | 0.0 | 0 | 0.0 | 7 | 15.9 |
| Relief | 9 | 50.0 | 10 | 61.5 | 4 | 42.9 | 0 | 0.0 | 23 | 52.3 |
| Joy | 16 | 77.3 | 10 | 69.2 | 3 | 42.9 | 0 | 0.0 | 29 | 65.9 |
| Guilt | 3 | 13.6 | 2 | 15.4 | 0 | 0.0 | 0 | 0.0 | 5 | 11.4 |
| Irritation | 0 | 0.0 | 2 | 23.1 | 1 | 14.3 | 1 | 50.0 | 4 | 9.1 |
| Embarrassment | 3 | 18.2 | 2 | 15.4 | 2 | 28.6 | 1 | 50.0 | 8 | 18.2 |
| Pride | 1 | 13.6 | 7 | 38.5 | 2 | 14.3 | 0 | 0.0 | 10 | 22.7 |
| Sadness | 8 | 36.4 | 5 | 46.2 | 6 | 71.4 | 2 | 100.0 | 21 | 47.7 |
| Fascination | 6 | 22.7 | 0 | 7.7 | 2 | 14.3 | 0 | 0.0 | 8 | 18.2 |
| Fear | 0 | 0.0 | 2 | 23.1 | 1 | 14.3 | 1 | 50.0 | 4 | 9.1 |
| Disgust | 0 | 0.0 | 0 | 0.0 | 0 | 0.0 | 0 | 0.0 | 0 | 0.0 |
| None | 1 | 4.5 | 0 | 0.0 | 0 | 0.0 | 0 | 0.0 | 1 | 2.3 |

Supplementary Table 5. Frequency distribution of “being moved” ratings in Study 2 (recalling memories vs. viewing Paintings in an Exhibition) and Study 3 (exploring the whole exhibition)

| **Study / task** | | **N** | **Rating** | | | | | | | | | | |
| --- | --- | --- | --- | --- | --- | --- | --- | --- | --- | --- | --- | --- | --- |
|  |  |  | **0** | **1** | **2** | **3** | **4** | **5** | **6** | **7** | **8** | **9** | **10** |
| Study 2 (recalling) | n | 46 | 0 | 0 | 1 | 3 | 0 | 1 | 5 | 5 | 7 | 9 | 15 |
|  | % | - | 0 | 0 | 2.2 | 6.5 | 0 | 2.2 | 10.9 | 10.9 | 15.2 | 19.6 | 32.6 |
| Study 2 (paintings) | n | 228 | 7 | 15 | 26 | 29 | 22 | 20 | 31 | 38 | 30 | 6 | 4 |
|  | % | - | 3 | 6.5 | 11.3 | 12.6 | 9.6 | 8.7 | 13.5 | 16.5 | 13 | 2.6 | 1.7 |
| Study 3 (exhibition) | n | 32 | 0 | 3 | 4 | 4 | 3 | 4 | 6 | 4 | 4 | 0 | 0 |
|  | % | - | 0 | 9.4 | 12.5 | 12.5 | 9.4 | 12.5 | 18.8 | 12.5 | 12.5 | 0 | 0 |

Supplementary Table 6. Descriptive statistics and regression summary of scales associated with being moved by paintings (Study 2)

| **Scale** | **Med-ian** | **IQR** | **Mean** | **SD** | **β** | **SE** | **95% CI** | **Shapiro–Wilk p** | **t** | **p (FDR)** |
| --- | --- | --- | --- | --- | --- | --- | --- | --- | --- | --- |
| Positive mood | 6 | 3.25 | 5.658 | 2.565 | 0.07 | 0.064 | [-0.056, 0.196] | 0.020 | 1.091 | 0.291 |
| Negative mood | 1 | 4 | 2.145 | 2.141 | 0.253 | 0.067 | [0.121, 0.385] | 0.043 | 3.76 | 0.000 |
| Happiness | 5 | 5 | 4.399 | 2.878 | 0.068 | 0.067 | [-0.062, 0.199] | 0.019 | 1.027 | 0.305 |
| Sadness | 1.5 | 3 | 2.263 | 2.349 | 0.326 | 0.062 | [0.204, 0.447] | 0.328 | 5.26 | 0.000 |
| Being moved | 5 | 4 | 4.947 | 2.515 | - | - | - | - | - | - |
| Arousal | 4 | 4 | 4.263 | 2.38 | 0.136 | 0.067 | [0.005, 0.267] | 0.047 | 2.037 | 0.05 |
| Meaning understood | 4 | 4 | 4.329 | 2.668 | 0.364 | 0.06 | [0.247, 0.481] | 0.072 | 6.087 | 0.000 |
| Engaged | 5 | 4.25 | 4.724 | 2.666 | 0.58 | 0.057 | [0.468, 0.693] | 0.852 | 10.107 | 0.000 |
| Expected gain | 4 | 5 | 3.833 | 2.83 | 0.416 | 0.064 | [0.289, 0.542] | 0.020 | 6.444 | 0.000 |
| Interest / curiosity | 6 | 4.25 | 5.575 | 2.744 | 0.404 | 0.065 | [0.277, 0.53] | 0.390 | 6.245 | 0.000 |
| Initial difficulty | 2 | 4.25 | 3.079 | 2.752 | -0.111 | 0.065 | [-0.239, 0.017] | 0.029 | -1.694 | 0.102 |
| Revisited | 4 | 6 | 4.285 | 3.45 | 0.216 | 0.071 | [0.077, 0.355] | 0.032 | 3.051 | 0.004 |
| Kindness / integrity | 3 | 5 | 3.491 | 2.938 | 0.284 | 0.062 | [0.163, 0.404] | 0.132 | 4.605 | 0.000 |
| Closeness | 3 | 6 | 3.697 | 3.123 | 0.33 | 0.063 | [0.206, 0.454] | 0.078 | 5.219 | 0.000 |
| Welcomed | 3 | 6 | 3.504 | 3.128 | 0.185 | 0.064 | [0.06, 0.31] | 0.042 | 2.905 | 0.006 |
| Emotional memory | 3 | 6 | 3.917 | 3.377 | 0.291 | 0.061 | [0.17, 0.411] | 0.181 | 4.743 | 0.000 |
| Cherished person | 2 | 6.25 | 3.303 | 3.316 | 0.266 | 0.065 | [0.139, 0.393] | 0.008 | 4.107 | 0.000 |
| Express care | 1 | 4 | 2.36 | 2.69 | 0.237 | 0.07 | [0.101, 0.374] | 0.166 | 3.416 | 0.001 |
| Hug someone | 1 | 4 | 2.206 | 2.638 | 0.204 | 0.069 | [0.069, 0.339] | 0.076 | 2.956 | 0.005 |
| Do something nice | 1 | 3 | 1.939 | 2.383 | 0.162 | 0.073 | [0.018, 0.305] | 0.054 | 2.207 | 0.035 |
| Relationship commitment | 1 | 3 | 1.825 | 2.526 | 0.159 | 0.072 | [0.018, 0.3] | 0.042 | 2.211 | 0.035 |

Supplementary Table 7. Regression summary of art features associated with being moved by paintings (Study 2)

| **Scale** | **n** | **%** | **β** | **SE** | **95% CI** | **Shapiro–Wilk p** | **t** | **p (FDR)** |
| --- | --- | --- | --- | --- | --- | --- | --- | --- |
| Color | 171 | 75 | -0.127 | 0.365 | [-0.842, 0.587] | 0.051 | -0.350 | 0.814 |
| Texture | 89 | 39 | -0.077 | 0.321 | [-0.706, 0.553] | 0.055 | -0.239 | 0.841 |
| Light & Shadow | 66 | 28.9 | 0.177 | 0.347 | [-0.503, 0.858] | 0.069 | 0.511 | 0.755 |
| Composition | 120 | 52.6 | 0.272 | 0.32 | [-0.355, 0.899] | 0.063 | 0.851 | 0.554 |
| Scale / Size | 54 | 23.7 | -0.464 | 0.376 | [-1.2, 0.272] | 0.036 | -1.235 | 0.359 |
| Detail | 72 | 31.6 | 0.461 | 0.351 | [-0.227, 1.149] | 0.037 | 1.313 | 0.334 |
| Lines | 40 | 17.5 | -0.193 | 0.42 | [-1.016, 0.631] | 0.053 | -0.458 | 0.755 |
| Content & Meaning | 91 | 39.9 | 1.425 | 0.312 | [0.813, 2.038] | 0.052 | 4.565 | 0.000 |
| Symbolism / Metaphor | 84 | 36.8 | 0.788 | 0.33 | [0.141, 1.434] | 0.037 | 2.388 | 0.055 |
| Story / Background | 24 | 10.5 | 0.039 | 0.515 | [-0.972, 1.049] | 0.050 | 0.075 | 0.940 |
| Personal Link | 60 | 26.3 | 1.273 | 0.353 | [0.58, 1.965] | 0.043 | 3.601 | 0.003 |
| Moral / Social Value | 28 | 12.3 | 0.877 | 0.475 | [-0.055, 1.809] | 0.022 | 1.845 | 0.155 |
| Familiarity | 37 | 16.2 | 0.683 | 0.425 | [-0.151, 1.517] | 0.045 | 1.605 | 0.220 |
| Surprise | 35 | 15.4 | -0.837 | 0.425 | [-1.67, -0.004] | 0.082 | -1.970 | 0.128 |
| Physical Context | 38 | 16.7 | 0.118 | 0.451 | [-0.767, 1.003] | 0.045 | 0.262 | 0.841 |
| Other | 0 | 0 | 0.638 | 1.356 | [-2.02, 3.296] | 0.049 | 0.471 | 0.755 |
| Moist Eyes | 3 | 1.3 | 3.326 | 1.336 | [0.707, 5.944] | 0.044 | 2.489 | 0.048 |
| Tears | 0 | 0 | - | - | - | - | - | - |
| Goosebumps | 23 | 10.1 | 1.103 | 0.523 | [0.077, 2.129] | 0.107 | 2.108 | 0.101 |
| Chills / Shivers | 19 | 8.3 | 0.566 | 0.565 | [-0.542, 1.674] | 0.071 | 1.001 | 0.469 |
| Warm Chest | 59 | 25.9 | 0.965 | 0.353 | [0.272, 1.658] | 0.079 | 2.730 | 0.027 |
| Chest Sensation | 75 | 32.9 | 0.963 | 0.351 | [0.276, 1.651] | 0.263 | 2.745 | 0.027 |
| Choked Up | 15 | 6.6 | 1.881 | 0.63 | [0.646, 3.115] | 0.096 | 2.986 | 0.018 |
| Lump in Throat | 29 | 12.7 | 2.231 | 0.476 | [1.299, 3.163] | 0.403 | 4.690 | 0.000 |
| Hard to Speak | 10 | 4.4 | 1.018 | 0.768 | [-0.487, 2.523] | 0.131 | 1.326 | 0.334 |
| Smile | 82 | 36 | 0.226 | 0.332 | [-0.425, 0.877] | 0.045 | 0.680 | 0.663 |
| Light / Buoyant | 34 | 14.9 | 0.726 | 0.442 | [-0.14, 1.592] | 0.032 | 1.644 | 0.220 |
| Refreshed / Energized | 56 | 24.6 | -0.445 | 0.375 | [-1.179, 0.29] | 0.076 | -1.187 | 0.369 |
| N/A | 36 | 15.8 | -1.858 | 0.463 | [-2.765, -0.951] | 0.119 | -4.016 | 0.001 |

Supplementary Table 8. Median ratings of scales across different types of moving events in autobiographical memories (Study 2)

| **Scale** | **Relationship** | **Critical life** | **Art-related** | **Nature-related** | **Overall** |
| --- | --- | --- | --- | --- | --- |
| Positive mood | 8 | 6 | 7 | 10 | 8 |
| Negative mood | 1 | 1 | 2 | 0 | 1 |
| Happiness | 9 | 5 | 6 | 10 | 8 |
| Sadness | 2 | 5 | 4 | 0 | 2 |
| Being moved | 8 | 9 | 9 | 10 | 9 |
| Arousal | 7 | 3 | 7 | 6 | 6.5 |
| Closeness | 8 | 8 | 7 | 8 | 8 |
| Welcomed | 7 | 4 | 3 | 8 | 6 |
| Emotional memory | 8 | 8 | 2 | 8 | 7 |
| Cherished person | 8 | 7 | 2 | 7 | 7 |
| Express care | 7 | 6 | 2 | 7 | 6 |
| Hug someone | 8.5 | 7 | 1 | 6 | 6.5 |
| Do something nice | 4 | 2 | 1 | 1 | 2 |
| Relationship commitment | 5 | 5 | 2 | 3 | 3 |

Supplementary Table 9. Frequency (%) of bodily sensations across different types of moving events in autobiographical memories (Study 2)

| **Scale** | **Relationship** | **Critical life** | **Art-related** | **Nature-related** | **Overall** |
| --- | --- | --- | --- | --- | --- |
| Moist Eyes | 27.8 | 44.4 | 63.6 | 28.6 | 39.1 |
| Tears | 16.7 | 55.6 | 36.4 | 0.0 | 26.1 |
| Goosebumps | 11.1 | 33.3 | 54.5 | 42.9 | 30.4 |
| Chills / Shivers | 16.7 | 0.0 | 27.3 | 28.6 | 17.4 |
| Warm Chest | 72.2 | 44.4 | 54.5 | 85.7 | 63.0 |
| Chest Sensation | 33.3 | 33.3 | 36.4 | 0.0 | 28.3 |
| Choked Up | 27.8 | 44.4 | 36.4 | 0.0 | 28.3 |
| Lump in Throat | 22.2 | 77.8 | 45.5 | 0.0 | 34.8 |
| Hard to Speak | 33.3 | 33.3 | 27.3 | 14.3 | 28.3 |
| Smile | 66.7 | 33.3 | 36.4 | 85.7 | 54.3 |
| Light / Buoyant | 27.8 | 11.1 | 9.1 | 42.9 | 21.7 |
| Refreshed / Energized | 27.8 | 11.1 | 18.2 | 71.4 | 30.4 |
| N/A | 0.0 | 0.0 | 9.1 | 0.0 | 2.2 |

Supplementary Table 10. Descriptive statistics and Spearman correlations between scales and being moved (Study 3)

| **Scale** | **Median** | **IQR** | **r** | **p(FDR)** |
| --- | --- | --- | --- | --- |
| Positive mood | 7 | 2.25 | 0.192 | 0.502 |
| Negative mood | 2 | 3 | -0.140 | 0.592 |
| General emotional arousal | 4 | 2.25 | 0.333 | 0.214 |
| Love | 4 | 3.25 | 0.308 | 0.244 |
| Happiness | 6.5 | 2 | 0.334 | 0.214 |
| Being shocked | 1 | 1.25 | 0.100 | 0.704 |
| Boredom | 1 | 2 | 0.035 | 0.865 |
| Anger | 0.5 | 1 | 0.151 | 0.578 |
| Playfulness | 4.5 | 3.5 | -0.123 | 0.634 |
| Sadness | 1 | 3 | 0.228 | 0.418 |
| Sudden insight | 3 | 3.25 | 0.072 | 0.795 |
| Anxiety | 1 | 2.25 | 0.063 | 0.798 |
| A sense of beauty | 7 | 2 | 0.203 | 0.491 |
| Pleasure | 6 | 2 | 0.400 | 0.114 |
| Being moved | 5 | 3.25 | - | - |
| A transformation | 2 | 2 | 0.303 | 0.244 |
| Changing something | 3 | 3 | 0.447 | 0.114 |
| Being thrilled | 3 | 3.25 | 0.252 | 0.394 |
| A sense of engagement | 5.5 | 2.5 | 0.230 | 0.418 |
| Tenderness | 3.5 | 3.25 | 0.483 | 0.114 |
| Compassion | 4 | 3.25 | 0.399 | 0.114 |
| Hope | 3 | 3 | 0.407 | 0.114 |
| Guilt | 0 | 2 | -0.182 | 0.509 |
| Shame | 0.5 | 2 | -0.167 | 0.540 |
| Remorse | 0 | 2.25 | 0.031 | 0.865 |

Supplementary Table 11. Descriptive statistics and regression estimates linking individual‑difference scales to ratings of being moved during Study 1 and the exhibition-exploration task in Study 2. EC = Empathic Concern; AF = Art Frequency (mean of three items assessing how often participants visit art venues, collect art, and read about art); AI = Art Interest; AK = Art Knowledge; *Bewegt* = “being moved” (German); *Gerührt* = “touched/deeply moved” (German).

| **Stu-dy** | **Scale** | **Medi-an** | **IQR** | **Scale Range** | **Outcome** | **β** | **SE** | **95% CI** | **t** | **p(FD-R)** |
| --- | --- | --- | --- | --- | --- | --- | --- | --- | --- | --- |
| 1 | EC | 12 | 2 | [4,16] | *Bewegt* | 0.12 | 0.20 | [-0.30, 0.54] | 0.59 | 0.92 |
|  |  |  |  |  | *Gerührt* | 0.02 | 0.23 | [-0.45, 0.50] | 0.10 | 0.92 |
|  | AF | 7 | 1 | [1,7] | *Bewegt* | -0.25 | 0.20 | [-0.66, 0.16] | -1.27 | 0.66 |
|  |  |  |  |  | *Gerührt* | -0.11 | 0.22 | [-0.58, 0.36] | -0.50 | 0.92 |
|  | AI | 45 | 18 | [11,77] | *Bewegt* | 0.36 | 0.20 | [-0.06, 0.79] | 1.78 | 0.43 |
|  |  |  |  |  | *Gerührt* | 0.39 | 0.23 | [-0.09, 0.87] | 1.70 | 0.43 |
| 2 | EC | 5.43 | 0.75 | [0,10] | Being Moved | 0.36 | 0.24 | [-0.13, 0.85] | 1.49 | 0.72 |
|  | AF | 2 | 1 | [1,7] |  | 0.18 | 0.31 | [-0.45, 0.81] | 0.57 | 0.94 |
|  | AI | 7 | 3.25 | [0,10] |  | 0.25 | 0.33 | [-0.41, 0.90] | 0.76 | 0.94 |
|  | AK | 3 | 3 | [0,10] |  | -0.07 | 0.33 | [-0.74, 0.60] | -0.21 | 0.94 |

Supplementary Table 12. Descriptive statistics and Spearman correlations (ρ) between individual‑difference scales and ratings of being moved during the autobiographical memory recall task in Study 2, and Study 3. EC = Empathic Concern; AF = Art Frequency (mean of three items assessing how often participants visit art venues, collect art, and read about art); AI = Art Interest; AK = Art Knowledge.

| **Study** | **Scale** | **Median** | **IQR** | **Scale Range** | **r** | **p(FDR)** |
| --- | --- | --- | --- | --- | --- | --- |
| 2 | EC | 5.43 | 0.75 | [0,10] | 0.34 | 0.08 |
|  | AF | 2 | 1 | [1,7] | -0.02 | 0.89 |
|  | AI | 7 | 3.25 | [0,10] | 0.11 | 0.63 |
|  | AK | 3 | 3 | [0,10] | -0.12 | 0.63 |
| 3 | EC | 7.5 | 2.5 | [0,10] | 0.24 | 0.68 |
|  | AF | 2.33 | 1 | [1,7] | -0.03 | 0.87 |
|  | AI | 8 | 2 | [0,10] | -0.14 | 0.68 |
|  | AK | 2 | 3.75 | [0,10] | 0.12 | 0.68 |
